# Linking Terpene Synthases to Sesquiterpene Metabolism in Grapevine Flowers

**DOI:** 10.3389/fpls.2019.00177

**Published:** 2019-02-21

**Authors:** Samuel Jacobus Smit, Melané Alethea Vivier, Philip Richard Young

**Affiliations:** Institute for Wine Biotechnology, Department of Viticulture and Oenology, Stellenbosch University, Stellenbosch, South Africa

**Keywords:** TPS, grapevine, chemotype, flower, sesquiterpene

## Abstract

Grapevine (*Vitis vinifera* L.) terpene synthases (VviTPS) are responsible for the biosynthesis of terpenic volatiles. Volatile profiling of nine commercial wine cultivars showed unique cultivar-specific variation in volatile terpenes emitted from grapevine flowers. The flower chemotypes of three divergent cultivars, Muscat of Alexandria, Sauvignon Blanc and Shiraz were subsequently investigated at two flower developmental stages (EL-18 and -26). The cultivars displayed unique flower sesquiterpene compositions that changed during flower organogenesis and the profiles were dominated by either (*E*)-β-farnesene, (*E,E*)-α-farnesene or (+)-valencene. *In silico* remapping of microarray probes to *VviTPS* gene models allowed for a meta-analysis of *VviTPS* expression patterns in the grape gene atlas to identify genes that could regulate terpene biosynthesis in flowers. Selected sesquiterpene synthase genes were isolated and functionally characterized in three cultivars. Genotypic differences that could be linked to the function of a targeted gene model resulted in the isolation of a novel and cultivar-specific single product sesquiterpene synthase from Muscat of Alexandria flowers (VvivMATPS10), synthesizing (*E*)-β-farnesene as its major volatile. Furthermore, we identified structural variations (SNPs, InDels and splice variations) in the characterized *VviTPS* genes that potentially impact enzyme function and/or volatile sesquiterpene production in a cultivar-specific manner.

## Introduction

Evolution has resulted in tremendous chemical diversity of floral scent within and across species. Terpene synthases (TPS) are responsible for the biosynthesis of terpenoids, a class of natural products consisting of more than 50,000 compounds in plants (Christianson, 2008; Osbourn and Lanzotti, 2009; Buckingham et al., 2015), of which ∼556 are known to contribute to floral scent (Knudsen and Gershenzon, 2006). A TPS typically catalyzes the final step in terpene biosynthesis with enzymes having the capacity to synthesize either a single terpene or multiple compounds (Christianson, 2017). This is mainly due to the complex mechanism of the enzyme; one of the most significant aspects being how the enzyme’s active site interacts with its substrate (Degenhardt et al., 2009). TPS substrate biosynthesis results from the head-to-tail coupling of C_5_ prenylated precursors, namely isopentenyl phosphate (IPP) and dimethylallyl phosphate (DMAPP), that are synthesized by the plastidial 2*C*-methyl-D-erythritol-4-phosphate (MEP) or cytosolic mevalonate (MVA) pathway (Bloch et al., 1959; Lichtenthaler, 1999; Rohmer, 1999). Although these pathways are compartmentalized, metabolic “crosstalk” has been shown to result in these precursors being transported between the plastids and cytosol (Piel et al., 1998; Adam et al., 1999; Jux et al., 2001; Bick and Lange, 2003; Hemmerlin et al., 2003; Schuhr et al., 2003). Regulation of these pathways has been shown to be spatial, temporal and/or diurnal, depending on the species and organ involved in biosynthesis (Dudareva and Pichersky, 2006). The C_10_ geranyl diphosphate (GPP) and C_15_ farnesyl diphosphate (FPP) substrates result in the biosynthesis of the majority of flower terpenes, namely mono- and sesquiterpenes, respectively (Davis and Croteau, 2000).

A TPS facilitates a complex biochemical cascade involving cyclizations and/or rearrangement of the substrate to form acyclic and cyclic terpenes. These cascades proceed through reactive intermediates, referred to as carbocations, that serve as branchpoints for specific trajectories in the chemical cascade. It is thus possible to group terpenes based on the similarity of carbocations/cascade required for biosynthesis (Allemann et al., 2007; Hare and Tantillo, 2016; Christianson, 2017). Although the crystal structures of mono- and sesquiterpene synthases have been elucidated (Lesburg, 1997; Starks, 1997; Caruthers et al., 2000; Rynkiewicz et al., 2001; Shishova et al., 2007; Gennadios et al., 2009), the exact path from substrate to terpene is not always known, or conclusively determined. Computational chemistry has proven useful in predicting these structures and the reaction mechanism that will result in terpenes under biologically relevant conditions (Allemann et al., 2007; Miller et al., 2008; Hess et al., 2011; Tantillo, 2011; Wedler et al., 2015;O’Brien et al., 2016).

Plant terpenes are typically studied for their ecological/biological roles which include pollinator attraction (Pichersky and Gershenzon, 2002), direct and indirect pest/pathogen/cellular defense (Köllner et al., 2008; Sabater-Jara et al., 2010; Zulak and Bohlmann, 2010; Lawo et al., 2011) and chemical signaling (Shen et al., 2000; Van Poecke et al., 2001; Köllner et al., 2008; Copolovici et al., 2012). Grapevine (*Vitis vinifera* L.) is a commercially important crop with an expanded *VviTPS* family consisting of 152 loci, of which 69 encode for putatively functional proteins (Martin et al., 2010). Grapevine terpenes have been mainly studied for their roles in modulating flavor and aroma profiles of grape berries and wine, with a particular focus on VviTPSs that synthesize terpenes imparting floral (e.g., linalool and limonene) and pepper (e.g., rotundone) aromas (Siebert et al., 2008; Skinkis et al., 2008; Wood et al., 2008; Matarese et al., 2013). The biological/ecological role of grapevine terpenes is, however, not well established, although a limited number of studies hold promise for identifying such roles. For example, the terpenes (*E*)-β-farnesene, (*E*)-β-caryophyllene and (*E*)-4,8-dimethyl-1,3,7-nonatriene were shown to act as semiochemicals for the phytophagous moth *Lobesia botrana*, a major pest in European vineyards (Tasin et al., 2005; Anfora et al., 2009; von Arx et al., 2011; Salvagnin et al., 2018). Also, cultivar-specific resistance toward phylloxera (*Daktulosphaira vitifoliae*) has been linked to root terpene biosynthesis (Lawo et al., 2011). A potential role in antioxidant protection in response to ultraviolet light has also been proposed for grapevine leaf terpenes (Gil et al., 2012).

Grapevine flowers show the most significant expression of *VviTPS* genes, compared to other organs in the grapevine gene atlas (Fasoli et al., 2012). A concordant emission of terpenes has been observed in a limited number of cultivars profiled for their flower volatile emissions (Buchbauer et al., 1994a,b, 1995; Martin et al., 2009; Barbagallo et al., 2014; Matarese et al., 2014). These results clearly showed that grapevine flowers have a unique transcriptional and biosynthetic capacity to produce and emit terpenes, with the majority of cultivars emitting mainly sesquiterpenes, even though the biological/ecological role(s) for domesticated grapevine flower terpenes remain to be established. Furthermore, the reported volatile profiles suggested that there are differences between cultivars, but it is difficult to directly compare the results from the different studies, given the variety of analytical techniques used to profile the grapevine flowers (Buchbauer et al., 1994a,b, 1995; Martin et al., 2009; Barbagallo et al., 2014; Matarese et al., 2014).

One of the aims of this study was to link terpenic profiles of the flower terpene emissions of a few selected, globally important commercial cultivars of grapevine, to functionally characterized *VviTPS* genes. Cultivar variations in terpene biosynthesis could be due to a variety of genetic and/or biochemical factors. To date 30 of the 69 putative *VviTPS* gene models (Martin et al., 2010) identified on the PN40024 reference genome (Jaillon et al., 2007) have been functionally characterized, of which 16 encode for sesqui- and 7 for mono-*TPS* genes. These 30 gene models are associated with 42 enzymes producing a broad range of terpenes and were isolated from a multitude of tissue types and cultivars (Lücker et al., 2004; Martin and Bohlmann, 2004; Martin et al., 2010; Drew et al., 2015). The reference genome revealed that the VviTPS family is greatly expanded, likely due to a complicated domestication history where the modern domesticated species shows greater diversity and heterozygosity than the ancient parents (Aradhya et al., 2003; Salmaso et al., 2004; Laucou et al., 2018). Crossing of distantly related parents, coupled with clonal propagation, have resulted in numerous heterozygous genotypes with their genetic diversity not reflected in the highly inbred, near homozygous reference genome (Da Silva et al., 2013; Roach et al., 2018; Minio et al., 2019). For example, a comparison between the reference genome and the Tannat cultivar revealed that 8–10% of genes are unshared, referred to as cultivar specific or “private” genes (Da Silva et al., 2013). Furthermore, these private genes contribute to cultivar specific phenotypes and account for the majority of uniquely expressed genes (Da Silva et al., 2013). More recently, the application of single cell sequencing technology revealed that the genome of Cabernet Sauvignon contains private genes not present in PN40024, Tannat, Nebbiolo or Corvina genomes (Minio et al., 2019) while a similar study in Chardonnay extended genotypic differences even further by showing the extent of structural variations within fifteen clones of this cultivar (Roach et al., 2018). Other approaches to identify structural variations between genotypes include the analyses of molecular markers, like nuclear microsatellites (nSSRs) or single nucleotide polymorphisms (SNPs), where evidence of extensive genotypic differences is shown (Aradhya et al., 2003; This et al., 2004; Ibáñez et al., 2009; Myles et al., 2011; Emanuelli et al., 2013; Picq et al., 2014; Nicolas et al., 2016; Laucou et al., 2018). A second focus of this study was therefore to understand the genetic factors that could determine how cultivar genotypes differ in terms of terpene biosynthesis.

Although the PN40024 reference genome is limiting when viewing genotypic variation, it still allowed for the generation of numerous expression datasets that can be mined to understand the VviTPS family. One of the most useful datasets is that of the grapevine gene atlas (Fasoli et al., 2012) which consists of 54 different organs and tissue types, comprehensively profiling gene expression throughout the plant. Unfortunately it underrepresents the *VviTPS* family due to the microarray probe design being based on computationally identified gene models of the CRIBI.v1 genome annotation (Jaillon et al., 2007; Forcato, 2010; Adam-Blondon et al., 2011; Grimplet et al., 2012; Adam-Blondon, 2014). The 152 *VviTPS*-like loci identified by Martin et al. (2010) and resultant manually corrected *VviTPS* gene models differ greatly from the 70 *VviTPS*-like genes of the CRIBI.v1 genes analyzed on the gene atlas. Furthermore, cross-hybridization of probes on the grapevine microarrays can be extensive leading to a high false discovery rate (Moretto et al., 2016). We addressed these limitations through *in silico* remapping of the microarray probes from the gene atlas to the curated *VviTPS* gene annotations (Martin et al., 2010), allowing for the identification of specific VviTPS expression patterns. Grapevine flowers showed an interesting expression pattern with subsequent volatile profiling of flowers from nine cultivars showing terpene volatile differences. We therefore aimed to explore the extent of genotypic differences in *VviTPS* genes, and their potential impact on terpene metabolism by linking the *in silico* expression analyses with functional characterization of selected *VviTPS* gene models. Gene models were characterized in three different cultivars with gene structure variations (i.e., SNPs and InDels) that could impact enzyme function in a cultivar-specific manner evaluated. The results obtained in this study, and known VviTPS functions mentioned earlier, were used to postulate on the carbocation intermediates commonly utilized in grapevine flower sesquiterpene biosynthesis. This resulted in the generation of a model for metabolic cascades involved in grapevine flower sesquiterpene biosynthesis as dictated by cultivar-specific roles of VviTPSs.

## Materials and Methods

### Sampling and Volatile Analysis of Grapevine Flower Material

Nine *V. vinife*ra cultivars, namely Chardonnay (CH), Chenin Blanc (CB), Muscat of Alexandria (MA), Pinot noir (PN), Pinotage (PI), Sauvignon Blanc (SB), Shiraz (SH), Viognier (VG), and Weisser Riesling (WR), were sampled at the pre-anthesis flower stage, corresponding to stage 18 of the modified Eichorn-Lorenz (EL) phenological stage classification system (Coombe, 1995). Six to eight flower clusters per cultivar were obtained from a mother block in the Stellenbosch area (33°57′33.50′′S, 18°51′38.09′′E), South Africa in a vineyard where the respective cultivars were planted in close geographical proximity. Samples were flash frozen with liquid nitrogen and stored at -80°C. Flower rachises were separated from the samples before flowers were homogenized and stored at -80°C for subsequent analyses.

In a subsequent season, sampling of MA, SB and SH flowers were performed at two distinct developmental stages, the EL-18 and EL-26 (flower bloom) stages. For this sampling, we randomly sampled four biological repeats consisting of six to eight flower clusters per repeat from the same vineyard as described before. All cultivars were sampled between 9 and 10 am on a single day for the respective stages during the 2015 flower season.

A method optimized for grape berry aroma compound analysis (Young et al., 2015) was adapted to analyze flower tissue. 10 mg (±10% SD) frozen tissue was weighed off directly into a 20 mL glass vial containing 2 mL tartrate extraction buffer (5 g/L tartaric acid, 2 g/L ascorbic acid, 8 mg/L sodium azide and 250 g/L NaCl). The deuterated standard Anisole-D_8_ (Sigma-Aldrich, United States), prepared in acetonitrile served as internal standard and was added to the buffer at a final concentration of 0.1 mg/L. Vials were sealed using a screw cap. Solid phase micro-extraction (SPME) of the vial head space (HS) was done using a 50/30 μm gray divinylbenzene/carboxen/polydimethylsiloxane (DVB/CAR/PDMS) fiber (Supelco, Bellefonte, PA, United States) that underwent pre-conditioning at 270°C for 60 min in the GC injection port according to the manufacturer specifications.

Sample vials were pre-incubated for 5 min at 45°C in the autosampler heating chamber. The heating chamber was maintained at 45°C and agitated at 250 rpm to allow for equilibration of compounds between the sample and headspace. The fiber was inserted through the septa and exposed to the analytes in the headspace for 10 min, while maintaining the agitation speed and temperature at 250 rpm and 45°C, respectively. Desorption of the analytes took place in the GC injection port for 5 min, where after, the fiber was maintained for 20 min in order to prevent any carryovers.

An Agilent 6890N gas chromatograph (Agilent, Santa Clara, CA, United States) system coupled to a CTC CombiPal Analytics auto-sampler and an Agilent 5975B inert XL EI/CI MSD mass spectrometer detector through a transfer line was used for the analyses. A Zebron 7HG-G009-11 ZB-FFAP capillary 55 column, 30 m × 250 ID μm, 0.25 μm film thickness, (Phenomenex, United States) was used. The desorption temperature for the analytes was 250°C for 5 min with a 10:1 split. Helium served as the carrier gas having an initial flow rate of 1 mL/min. Initial oven temperature was maintained for 2 min at 40°C, followed by a linear increase of 10°C/min to a final temperature of 240°C which was held for an additional 2 min.

Authentic standards for identification and quantification of volatiles were purchased from Sigma-Aldrich, United States for (+)-valencene (≥70%), (*E*)-β-farnesene (≥90%), β-caryophyllene (≥80%), and α-humulene (≥96%). Stock solutions of the standards were prepared in methanol. A calibration curve was prepared in 2 mL tartrate buffer as described above containing 0.1 mg/L Anisole-D_8_ as internal standard.

The Qualitative Analysis package of MassHunter Workstation software (Agilent, Santa Clara, CA, United States) was used to visualize extracted ion chromatograms (IEC) using the cumulative response of the following masses: 41 and 55 for (*E*)-2-hexenal; 70 and 116 for the internal standard; 93, 161 and 189 for sesquiterpenes. IEC chromatogram peak areas were integrated using default parameters and normalized to the area of the internal standard. Compounds were identified using authentic standards, when available, and the Wiley 275 and NIST14 mass spectral libraries. Concentrations were determined according to the calibration curve of the respective authentic standards. Where an authentic standard was not available, we determined compound concentrations semi-quantitatively using the (+)-valencene standard curve.

### *In silico* Expression Pattern and Phylogenetic Analysis for the *VviTPS*-a Gene Family

Manual curations for the *VviTPS* gene family (Martin et al., 2010) were incorporated in the recently released 12X.v2 genome assembly and accompanying VCost.v3 (V3) annotation (Canaguier et al., 2017) and are referred to accordingly. We aimed to supplement the existing compendium of expression data (Moretto et al., 2016), generated using the CRIBI.V1 annotation, as described below.

Putatively functional *VviTPS* genes (Martin et al., 2010) were evaluated for their expression patterns in the grapevine gene atlas [GEO Accession GSE36128 (Fasoli et al., 2012)]. Probe sequences for the NimbleGen 090918 *Vitis vinifera* exp HX12 array (NCBI GEO Acc. GPL13936) were retrieved from the GEO database (Edgar et al., 2002) followed by analysis of probe binding ambiguity using BLAST homology with cut-off parameters that allowed for two sequence miss matches of the full-length probe sequence as aligned to the *VviTPS* gene models. RMA normalized expression values of the re-mapped probes were used to analyze the expression patterns with the clustermap function of the Seaborn package in Python (version 3.5.3).

VviTPS-a members (Lücker et al., 2004; Martin et al., 2009, 2010) were compared through multiple sequence alignments (MSAs) of derived protein sequences. CLC Main Workbench 7 (CLC Bio-Qiagen, Denmark) was used to perform MUSCLE alignments followed by phylogenetic tree construction using Maximum Likelihood Phylogeny with UPGMA as construction method, Jukes Cantor as substitution model and 100 bootstrap replicates.

### Isolation and Characterization of *VviTPS* Genes

Total nucleic acids were extracted from the MA, SB, and SH cultivars using the method described in Reid et al. (2006). RNA was purified and gDNA removed by on-column DNase I treatment using the Bioline Isolate II Plant RNA kit (Celtic Molecular Diagnostics, South Africa). RNA integrity was assessed on an agarose gel followed by cDNA synthesis using the ImPromII Reverse Transcription System (Promega, United States). Primers were designed with restriction digestion sites to facilitate directional cloning (Supplementary Table 1) using predicted cDNA sequences for *VviTPS* gene models described by Martin et al. (2010), as available on FLAGdb++ (Dèrozier et al., 2011). PCR reactions were performed using Phusion High Fidelity DNA polymerase (Thermo Fisher Scientific, United States). PCR products of expected sizes were purified from an agarose gel using the Qiagen Gel Extraction kit (Qiagen, United States) and A-tailed by incubation with the TaKaRa ExTaq proof-reading polymerase. A-tailed PCR products were ligated into a pGEM-T Easy vector (Promega, United States), transformed into chemically competent *Escherichia coli* and verified through bi-directional sequencing (Central Analytical Facility, Stellenbosch University, South Africa).

Isolated genes were sequenced and named according to the grapevine nomenclature standard for *V. vinifera* L. (Vviv) (Grimplet et al., 2014) with the gene model numbers used in the VCost.v3 annotation (Canaguier et al., 2017) preceded by the cultivar abbreviations for Muscat of Alexandria (MA), Sauvignon Blanc (SB), and Shiraz (SH): *VvivMATPS01* (MK100068), *VvivSBTPS01* (MK100069), *VvivSBTPS02* (MK100070), *VvivMATPS10* (MK100071), *VvivMATPS27* (MK100072), *VvivSHTPS27* (MK100073), *VvivMATPS28* (MK100074), *VvivSHTPS01* (MK100075), *VvivMATPS02* (MK100076), *VvivSHTPS02* (MK100077), *VvivSBTPS10* (MK100078), *VvivSHTPS10* (MK100079), *VvivSBTPS27* (MK100080), *VvivSBTPS28a* (MK100081), *VvivSBTPS28b* (MK100082), *VvivSHTPS28* (MK100083). Details regarding the specific cultivar clones are included in the above GenBank accessions.

Sequence analysis of gene isolates was performed using the CLC Main Workbench 7 (CLC Bio-Qiagen, Denmark) by searching for the presence of an open reading frame (ORF). Gene structures were predicted using Splign (Kapustin et al., 2008) with genomic sequences of target gene models (retrieved from FLAGdb++) used as the reference. Gene structures were visualized using the Gene Structure Display Server (Hu et al., 2015). Derived protein sequences were used to identify the N-terminal RRx_8_W and C-terminal DDxxD and NSE/DTE motifs described to be characteristic of *TPS* genes (Bohlmann et al., 1998; Aubourg et al., 2002) using the FIMO tool of the MEME suite (Bailey et al., 2009; Grant et al., 2011). The CLC Main Workbench 7 (CLC Bio-Qiagen, Denmark) was used to generate all MSAs.

### *In vivo* Heterologous Expression of *VviTPS* Cultivar Variants in Yeast and Volatile Profiling

Sub-cloning of putatively functional *VviTPS* genes from pGem-T Easy (Promega, United States) vectors were performed through restriction enzyme excision and ligation with T4 ligase (Promega, United States) into an inducible yeast expression vector harboring a *GAL1* promoter and the *URA3* auxotrophic marker. Expression vectors were transformed into *E. coli*, followed by PCR screening for positives and subsequent plasmid isolations. Positive expression vectors were linearized with *Apa*I (Thermo Fisher Scientific, United States) and transformed into *Saccharomyces cerevisiae* strain GT051 using the TRAFO method (Gietz and Woods, 2002). The GT051 strain was modified from the Thomas and Rothstein (1989) W303a strain to increase the metabolic flux for the FPP terpene precursor by over-expression of a truncated *HMG1* (SGD:S000004540) and an *IDI1* (SGD:S000006038) gene. Yeast transformants were plated on modified TRAFO synthetic drop-out (SD) plates (Gietz and Woods, 2002) containing 2% (w/v) glucose and the amino acids adenine, leucine and uracil omitted for auxotrophic selection. Putative yeast transformants were verified by colony PCR.

Synthetic complete (SC) media (Gietz and Woods, 2002) was supplemented with MgSO_4_ to a final Mg^2+^ concentration of 5 mM and buffered to pH 6 using citrate-phosphate buffer. Pre-cultures of the respective yeast transformants were prepared in SC media with glucose (2% w/v) as a carbon source. Cells were harvested through centrifugation and washed with sterile water. *TPS*-expression was induced in sealed 20 mL GC-vials containing 5 mL SC media with galactose (2% w/v) as carbon source. Assays were performed in triplicate (three cultures per positive transformant). The starting optical density (OD) was 0.7 at 600 nm. After 16 h of induction at 30°C with shaking, vials were placed at 4°C for 1 h before analysis. A 1 mL mixture of natamycin (Delvocid at 2 mg/mL in 0.1 M NaOH) and the internal standard Anisole-D_8_, prepared in acetonitrile at 50 μg/L final concentration, was added to each vial by piercing the vial septa using a sterile syringe. Delvocid was added to arrest biomass production, allowing for normalization to the internal standard.

HS-SPME-GC-MS was conducted using the same fiber, column, chromatograph and mass spectrometer detector as described before. The fiber was inserted through the septa and exposed to the analytes in the headspace for 20 min, while maintaining the agitation speed and temperature at 250 rpm and 35°C, respectively. Desorption of the analytes took place in the GC injection port where after the fiber was maintained for 20 min in order to prevent any carryovers. Desorption temperature for the analytes was 250°C for 5 min with a 10:1 split. Helium served as carrier gas with an initial flow rate of 1 mL/min. Initial oven temperature was maintained for 2 min at 40°C, followed by a linear increase of 10°C/min to a final temperature of 240°C which was held for an additional 2 min. The total run time was 24 min and the transfer line temperature 250°C. Calibration curves prepared in SC media, using the standards described earlier, were used for quantification and compound identification in combination with the Wiley 275 and NIST14 mass spectral libraries. Chromatograms were analyzed as described earlier.

### Transient Expression in *Nicotiana benthamiana*

Putative *VviTPS* genes were cloned into pDONR-Zeocin, using the 2-step PCR protocol to add attB sites, followed by an overnight BP reaction as described in the product manual (Thermo Fisher Scientific, United States). Entry clones were transformed into electrocompetent *E. coli* and colonies confirmed to be positive through sequencing. Expression clones were created using the pEAQ-HT-DEST1 vector (Sainsbury et al., 2009; Peyret and Lomonossoff, 2013) by performing an overnight LR reaction, followed by transformation into *E. coli* as above and restriction enzyme digestion of plasmids to confirm positive colonies. Clonases for Gateway cloning and the pDONR-Zeocin vector were purchased from Thermo Fisher Scientific, United States.

Destination vectors were transformed into electrocompetent *Agrobacterium tumefaciens* GV3101 and plated on LB plates with 30 μg/mL gentamycin, 50 μg/mL kanamycin, and 50 μg/mL rifampicin. Transient expression and volatile analysis in *N. benthamiana* was performed according to the method described by Bach et al. (2014) with minor adaptations: Overnight cultures were washed thrice with 0.9% (w/v) saline solution and resuspended to a final OD_600_ of 0.6 using MMA buffer [10 mM 2-[*N*-morpholino]ethanesulfonic acid (MES) pH 5.6, 10 mM MgCl_2_, 200 μM acetosyringone] instead of water. Resuspended cultures were incubated for 1 h at room temperature before infiltration. Two fully expanded leaves per plant were infiltrated in triplicate. Mock infiltrations with MMA buffer and non-infiltrated wild type plants served as controls. Qualitative analysis and compound identification were performed with the GC-MS instrument, software and (*E*)-β-farnesene analytical standard described earlier.

### Southern and Northern Blot Analysis of *VviTPS10*

A DIG probe targeting *VviTPS10* was obtained through PCR amplification of an 862 bp internal region of the coding sequence followed by DIG labeled as described in the DIG Application Manual for Filter Hybridization (Roche, Germany) and diluted to 8.2 ng/mL in DIG Easy Hyb. The same probe solution was used for both Southern and Northern blotting at the appropriate temperatures described in the DIG Application Manual for Filter Hybridization (Roche, Germany).

For Southern blot analysis genomic DNA was isolated from MA, SB, and SH using the method described by Lovato et al. (2012), followed by single digests of 10 μg gDNA using *Bam*HI, *Eco*RI and *Xba*I restriction enzymes (Thermo Fisher Scientific, United States). Southern blotting was performed as described in the DIG Application Manual for Filter Hybridization (Roche, Germany).

Total RNA was isolated from ±100 mg tissue for EL-18 and EL-26 stages from MA, SB, and SH using the method described by Reid et al. (2006). RNA was selectively purified using the RNeasy Mini kit (Qiagen) according to the RNA clean-up protocol described in the product manual. RNA samples were separated on a 1.2% formaldehyde agarose (FA) gel followed by Northern blot analysis according to the DIG Application Manual for Filter Hybridization (Roche, Germany).

### Biosynthetic Network of VviTPS and Heterologous Sesquiterpenes

VviTPS enzymes that have reported heterologous function (Lücker et al., 2004; Martin et al., 2009, 2010; Drew et al., 2015) along with enzymes isolated in this study were used to construct a virtual interaction network using Cytoscape (Version 3.4) (Shannon et al., 2003), available from http://www.cytoscape.org/. VviTPS enzymes were used as source nodes with their associated volatiles serving as target nodes, connected by an edge. Edges were weighted as a major volatile when their percentage contribution was greater than 10% with all volatiles contributing less then 10% deemed a minor volatile. Source nodes were colored according to the likely carbocation intermediate used in the majority of volatiles from the respective enzymes. We referred to Bülow and König (2000); Davis and Croteau (2000); Tantillo (2011); Miller and Allemann (2012); Wedler et al. (2015), and Durairaj et al. (2019) to predict the likely carbocation intermediate involved.

## Results

### *In silico* Expression Patterns of *VviTPS* Genes

Available *VviTPS* gene models (Martin et al., 2010) were re-assessed by re-mapping of probes-to-genes, as annotated on FLAGdb++ (Dèrozier et al., 2011), followed by expression pattern identification. The 69 putatively functional *VviTPS* gene models (predicted pseudo- and partial genes not considered for probe-to-gene remapping) (Martin et al., 2010) were re-analyzed to generate a network model (Supplementary Data Sheet 1). It was observed that probes often cross-hybridize with multiple *VviTPS* gene models, highlighting the close relatedness within the gene family. *In silico* remapping revealed a total number of 306 probes binding to the 69 putative *VviTPS* genes, with only 133 of these probes binding uniquely to a single gene model (Supplementary Figures 1A,B). Of these probes, only eight gene models showed the expected four probes per gene. The remaining probes had probe-to-gene binding ambiguity ratios between 1:2 and 1:6.

Using the remapped probe sets, *in silico* expression analysis was performed using the grapevine gene atlas. The *VviTPS* mapping provided for the NimbleGen 090918 *Vitis vinifera* exp HX12 platform can, however, also be used to view *VviTPS* expression for all experiments available in the GPL13936 platform.

**FIGURE 1:**
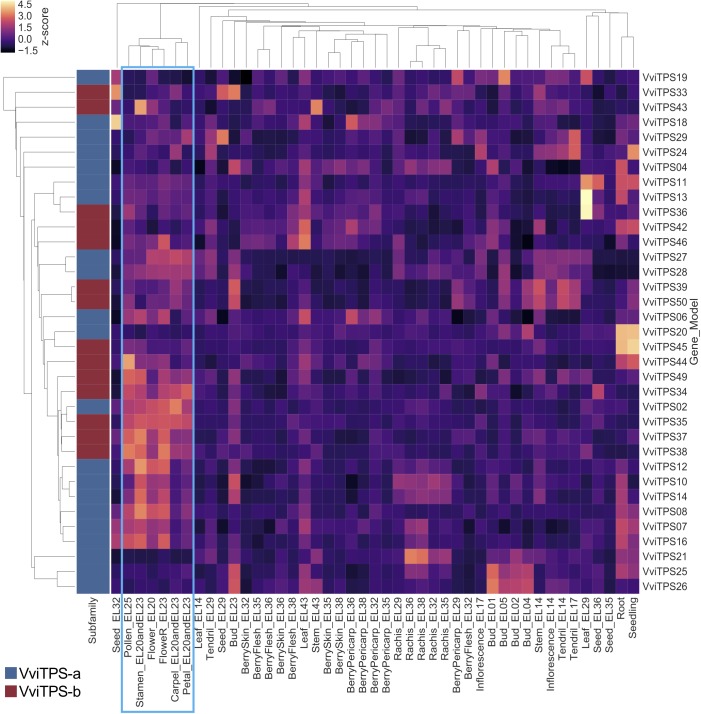
Per gene averaged expression of unambiguous probes for the VviTPS-a and-b subfamilies. Flowering stages and organs are highlighted by the blue square.

Global *VviTPS* expression was assessed by looking at all the probes individually. Two expression hotspots were identified, shown in the red and blue squares of Supplementary Figure 2. The blue square represented organs undergoing initial differentiation from budburst (EL-14) up to inflorescence establishment (EL-17), and include probes associated with mainly the VviTPS-a and -b subfamilies. The second hotspot (red square) showed high *VviTPS* expression in flower tissues from early bloom (EL-20) to full-bloom (EL-25), with the majority of probes also associated with VviTPS-a and -b subfamilies. Gene specific patterns were subsequently calculated by averaging all probes that bind uniquely to *VviTPS*-a and -b transcripts, illustrated in Figure 1. Only 35 of the 49 putatively functional VviTPS-a and -b members could be considered for Figure 1, with the remaining members represented only by ambiguously binding probes. A differential expression pattern for male and female flower organs was observed with *VviTPS-a* members (VviTPS07, -08, -10, -12, -14, and -16) showing greater expression in male parts while significantly lower expression in female parts. VviTPS27 and -28 showed the inverse with higher relative expression in female parts. The hotspot associated with inflorescence development (EL-14 to -17) was much less pronounced when probes are averaged, while the high relative expression at flower anthesis was still evident. In combination, the two approaches (the per probe and gene-averaged expression clustermaps) showed that VviTPS-a and -b subfamilies were highly expressed in floral organs with a differential pattern between pre- and full-bloom stages, suggesting that mono- and sesquiterpene biosynthesis could be upregulated during flower organogenesis.

### Profiling of Grapevine Flower Chemotypes

A selection of nine cultivars formed part of an initial screen to evaluate the formation of mono- and sesquiterpenes at flowering. Volatile analysis of flower samples at EL-18 stage of these cultivars (presumed to be the *VviTPS* transcriptional transition point from pre-bloom to bloom and including genes from hotspots identified in Figure 1 and Supplementary Figure 2) revealed that the cultivars differed significantly in terms of volatile content and composition, and that the majority of compounds present were sesquiterpenes (Supplementary Figure 3). (*E*)-2-hexenal was present at high concentrations for all cultivars along with heptadecene, tridecanone, eicosene, and 2-pentadecanone alkanes, at low abundance. Hierarchical clustering of the sesquiterpene volatiles identified cultivar differences in the chemotypes (Supplementary Figure 3) and identified the volatiles driving the differentiation. Two main clusters were identified with (*E,E*)-α-farnesene, (+)-valencene and its rearrangement 7-epi-α-selinene consistently present in all cultivars, except for CH and PI which lacked the latter two and produced (*E,E*)-α-farnesene as the major volatile. SB and SH were therefore selected as white and red varieties to represent this common chemotype with MA selected due to its unique chemotype, dominated by (*E*)-β-farnesene.

**FIGURE 2:**
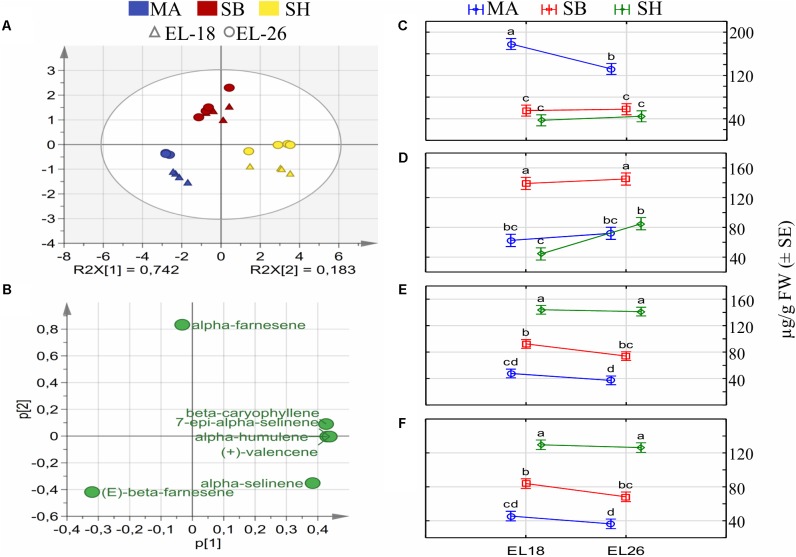
PCA scores **(A)** and loadings **(B)** of sesquiterpenes that drive differentiation between the cultivars Muscat of Alexandria (MA), Sauvignon Blanc (SB), and Shiraz (SH) at two phenological stages (EL-18 and EL-26). MANOVA of (*E*)-β-farnesene **(C)**, (*E,E*)-α-farnesene **(D)**, (+)-valencene **(E),** and 7-epi-α-selinene **(F)** shows statistically significant differences between cultivars and phenological stages of the most abundant sesquiterpenes emitted by grapevine flowers.

In-depth profiling of these three cultivars at two phenological stages were performed to expand on the different compositional ratios observed in the initial nine cultivar screen (Supplementary Figure 3). We identified a total of 12 flower sesquiterpenes with seven, namely β-caryophyllene, α-humulene, (*E*)-β-farnesene, (+)-valencene, α-selinene, 7-epi-α-selinene and (*E,E*)-α-farnesene consistently present in all three cultivars, regardless of flower stage (Supplementary Table 2). Chromatograms illustrating the volatile differences for these three cultivars can be viewed in Supplementary Figure 4. (+)-Aromadendrene, β-selinene, (*E*)-β-caryophyllene and (*Z,E*)-α-farnesene were emitted at low levels in a cultivar and/or stage specific manner, as shown in Supplementary Table 2. Multivariate data analysis tools were applied to identify variables that explain the variation observed between cultivars. Firstly, we used unsupervised principal component analysis (PCA) of the sesquiterpene volatiles, shown in Figure 2A,B. Cultivar sesquiterpene composition was shown to be the main driver for differences, contributing to 74.2% in the first component while stage differences explained 18.3% of the variation as the second component. The loadings plots (Figure 2B) was subsequently used to identify the volatiles that impart the most variation to the dataset with a multivariate analysis of variance (MANOVA) of these volatiles showing the extent of statistically significant differences between cultivars and/or stages (Figure 2C–F).

*(E*)-β-farnesene (Figure 2C) was significantly different for MA, compared to SB and SH. Furthermore, a significant difference for MA was observed between stages with 74% higher (*E*)-β-farnesene emission at EL-18 relative to EL-26. SB and SH produced (*E*)-β-farnesene at similar levels, regardless of phenological stage but at a concentration at least three times lower than MA. SB emitted (*E,E*)-α-farnesene (Figure 2D) as major volatile at near identical levels in both phenological stages. (*E,E*)-α-farnesene levels were significantly lower in MA and SH (ranging between 31 and 59%) relative to SB for both stages. However, SH showed an 85% relative increase for (*E,E*)-α-farnesene from EL-18 to EL-26. (+)-valencene (Figure 2E) and its rearrangement, 7-epi-α-selinene, (Figure 2F) had near identical emission levels that were statistically different between the cultivars, but not between stages within a cultivar. (+)-Valencene was the major volatile of SH. In summary, the results showed that the three cultivars each produced a specific major sesquiterpene and that their emission compositions changed between the EL-18 and EL-26 stages. The compositional changes were minor between cultivars, and within a cultivar, as flower organogenesis progressed. However, the presence/absence for the minor sesquiterpenes (+)-aromadendrene, β-selinene, (*E*)-caryophyllene and (*Z,E*)-α-farnesene contributed significantly to the cultivar- and stage-specific chemotypes.

### Selection of VviTPS-a Genes for Comparative Functional Characterization

**FIGURE 3:**
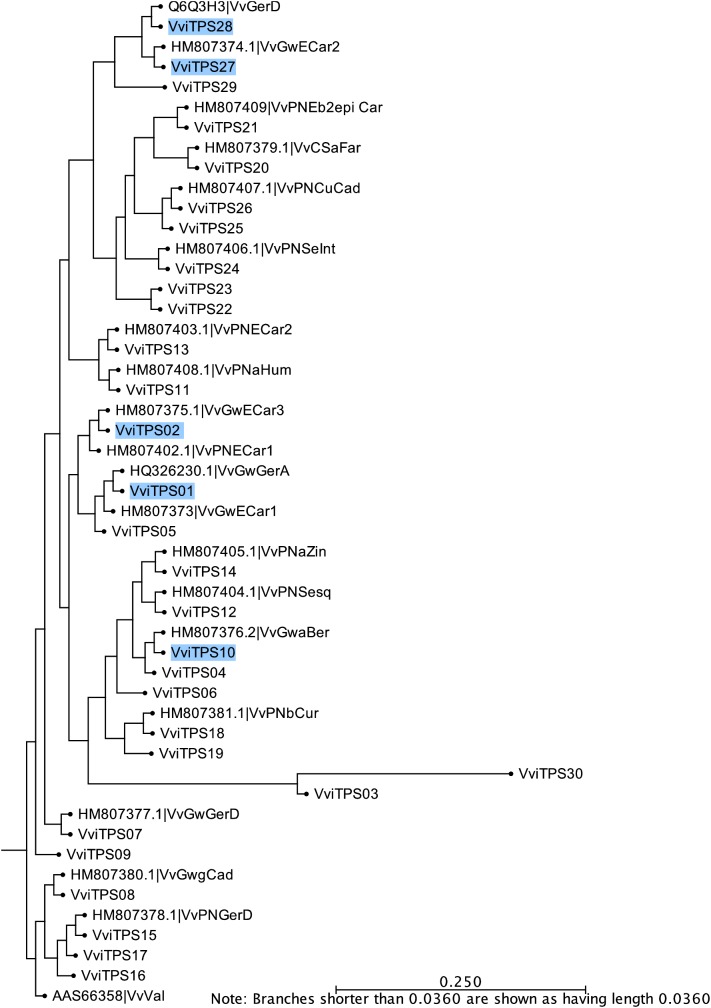
Phylogenetic tree of the VviTPS-a subfamily. Gene models targeted in the study are indicated by the blue circles.

Protein sequences derived from the predicted gene models showed subtle differences to the protein sequences of isolated and functionally characterized, illustrated in Figure 3. For example, five (*E*)-β-caryophyllene synthases, from two different cultivars, are associated with four different gene models (VviTPS02, -02, -13, and -27) (Martin et al., 2010), and although of similar function form distinctly different clades on the phylogenetic tree.

To investigate the extent of the genotypic variations and their potential impact on cultivar specific chemotypes we selected five candidate gene models (highlighted in blue in Figure 3). The *VviTPS01* gene model has been associated with two different functional sesquiterpene synthases, namely VvGwECar1 and VvGwGerA, producing (*E*)-β-caryophyllene and Germacrene A, respectively (Martin et al., 2010). This gene model also had a high number of ambiguously binding probes (Supplementary Data Sheet 1), suggesting that multiple variants or closely related genes exist. *In silico* expression patterns of the probes associated with *VviTPS01* furthermore show high relative expression in flowering tissue. *VviTPS02* and *VviTPS27* were dissimilar to *VviTPS01* on a sequence level but both were associated with functional enzymes, VvGwECar3/VvPnECar1 and VvGwECar2, respectively, producing (*E*)-β-caryophyllene as major product (Martin et al., 2010). *VviTPS10* was chosen due to its associated functional enzyme, VvGwaBer, producing (*E*)-β-farnesene as a minor secondary product. Twelve probes bound to this gene model, with only one binding unambiguously. Expression patterns for *VviTPS10* probes showed high relative expression in flowers. *VviTPS28* is associated with VvGerD (Lücker et al., 2004), which was characterized before the design of the microarray, resulting in four unique probes for the gene model (Supplementary Data Sheet 1). Furthermore, *VviTPS28*, along with *VviTPS27* showed high expression in both inflorescence and flower bloom stages (Supplementary Figure 2).

### Analysis of Isolated VviTPS-a Gene Sequences

**FIGURE 4:**
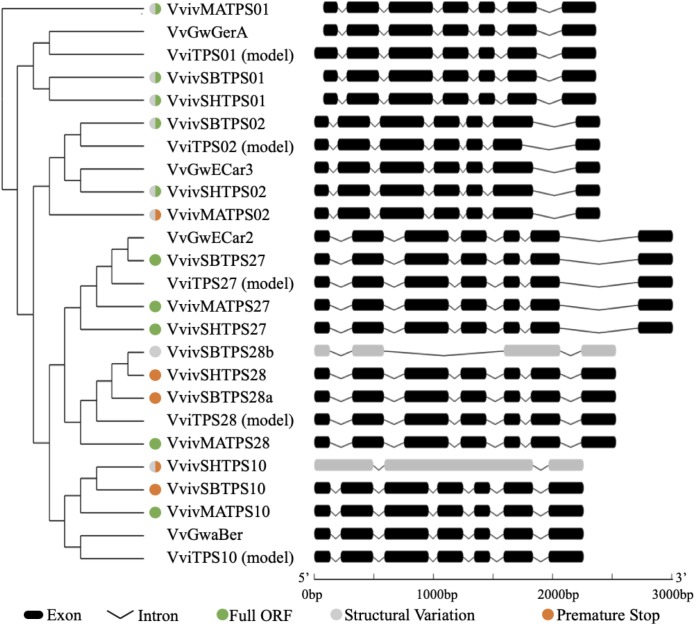
Structural organization of isolated *VvivTPS* cultivar variants compared to the reference gene model and associated functional genes. The dendrogram reflects maximum likelihood phylogeny using MUSCLE aligned coding sequences. Structural variations to the reference sequence, sequences with premature stop codons and those with a full-length open reading frame are indicated by the colored circles.

Sequenced isolates were compared to the predicted gene model and existing characterized genes mentioned earlier. This comparison revealed sequence and structural variations that potentially impact gene function, illustrated in Figure 4. *VvivTPS01 -02* isolates differed in gene structure to the gene model but contained a full length ORF and were therefore deemed putatively functional. The most prevalent cause for loss of function was due to SNPs that result in a premature stop codon. In addition to a premature stop we observed intron retention for *VvivSHTPS10*. Curiously PCR amplification with VviTPS28 primers resulted in two amplicons for SB with the second amplicon, *VvivSBTPS28b*, not being of the expected size. Gene sequencing results suggest that it is a partial duplicate of *VvivSBTPS28a*. *VvivSBTPS28b* maintained exons one and two, compared to the full-length sequence of *VvivSBTPS28b*, with a 596 nucleotide deletion resulting in the loss of exons three, four and a short part of exon five which shifted the start position for exon five. The intron between exons five and six was also retained. This isolate, however, has higher sequence homology to the SH variant than the SB variant.

Protein sequences were derived for the genes with a predicted full-length ORF and compared to that of the gene model (i.e., reference sequence) and its associated functional proteins (Lücker et al., 2004; Martin et al., 2010). These results are available in Supplementary Table 3 and the MSAs in Supplementary Data Sheet 2. VvivMATPS10 showed extensive sequence differences to both the reference sequence and VvGwaBer, with 37 of the 50 missense mutations located in the catalytic region of the enzyme. An amino acid deletion in the catalytic site was also observed.

### Heterologous Expression and Functional Characterization of VviTPS-a Cultivar Variants

Genes with full length ORFs were expressed *in vivo* using a heterologous yeast system with the percentage contribution of the observed volatiles reported in Table 1. Although, putatively functional, VvivSHTPS01 and VvivSBTPS27 produced no detectable volatiles and were therefore considered non-functional *in vivo*.

**Table 1:** Percentage contribution of volatiles produced through *in vivo* expression of VvivTPS cultivar variants.

	VvivSB-TPS01	VvivMA-TPS01	VvivSB-TPS02	VvivMA-TPS10	VvivSH-TPS27	VvivMA-TPS27	VvivMA-TPS28
β-Elemene	6.2%	5.5%	–	–	–	–	–
(*E*)-β-Caryophyllene	–	–	**100.0%**	–	**69.1%**	**63.7%**	–
(*E*)-β-Farnesene	–	–	–	**100.0%**	–	–	–
α-Humulene	5.3%	7.4%	–	–	–	–	–
β-Selinene	–	–	–	–	22.9%	24.2%	–
γ-Selinene	6.3%	–	–	–	–	–	–
Germacrene D	–	–	–	–	2.7%	3.7%	**56.4%**
β-Selinene	16.4%	15.1%	–	–	–	–	–
α-Selinene	**38.1%**	**49.1%**	–	–	–	–	–
Camphene	–	–	–	–	–	–	12.1%
γ-Cadinene	–	–	–	–	1.1%	1.6%	17.6%
α-Amorphene	–	–	–	–	–	–	13.9%
Germacrene A	23.6%	20.0%	–	–	0.9%	1.4%	–


*Major volatiles are shown in bold.*

*Agrobacterium* mediated transient expression of VvivMATPS10 confirmed functionality *in planta* as a single product enzyme synthesizing (*E*)-β-farnesene (Supplementary Figure 5).

### Genomic Localization and Flower Expression of VviTPS10

The dominance of (*E*)-*β*-farnesene in MA and the unique heterologous function of VvivMATPS10 prompted further inspection. *In silico* analysis showed that the *VviTPS10* gene model shared multiple probes with other *VviTPS* genes (Supplementary Data Sheet 1), suggesting that genes with high homology to *VviTPS10* are present in the PN40024 genome. Southern blot analysis confirmed the presence of *VviTPS10* homologs in all three cultivars, with three separate restriction enzyme digests of gDNA from MA, SB and SH (Supplementary Figure 6). Multiple hybridization patterns in the 4 to 1.9 kB regions, were observed, indicating the presence of numerous homologous genes within a cultivar.

**FIGURE 5:**
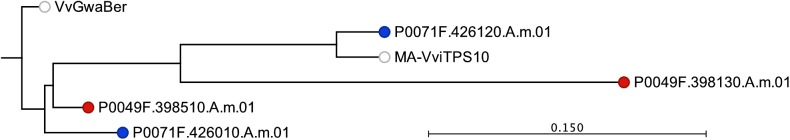
Maximum likelihood phylogeny of proteins homologous to VvivMATPS10 and VvGWaBer identified on two different primary contigs (red and blue nodes) of the phased-diploid Cabernet Sauvignon assembly.

We performed a preliminary analysis of the *VviTPS10* locus using the phased-diploid assembly and annotation of Cabernet Sauvignon (Chin et al., 2016; Minio et al., 2019). Two contigs, containing four homologous genes, were found with sequence phylogeny to *VvivMATPS10* and *VvGwaBer* and the four putative *VviTPS10*-like regions shown in Figure 5. The Cabernet Sauvignon *VviTPS10*-like genes are located on two different primary contigs with this shared location reflected in their phylogenetic grouping. Determining the expression of VvivMATPS10 was therefore not possible using quantitative PCR. Preliminary Northern blot analysis, however, suggested that *VviTPS10* is expressed in MA at both flower stages (Supplementary Figure 7).

### Proposed Carbocation Cascades Involved in Flower Chemotypic Differences

**FIGURE 6:**
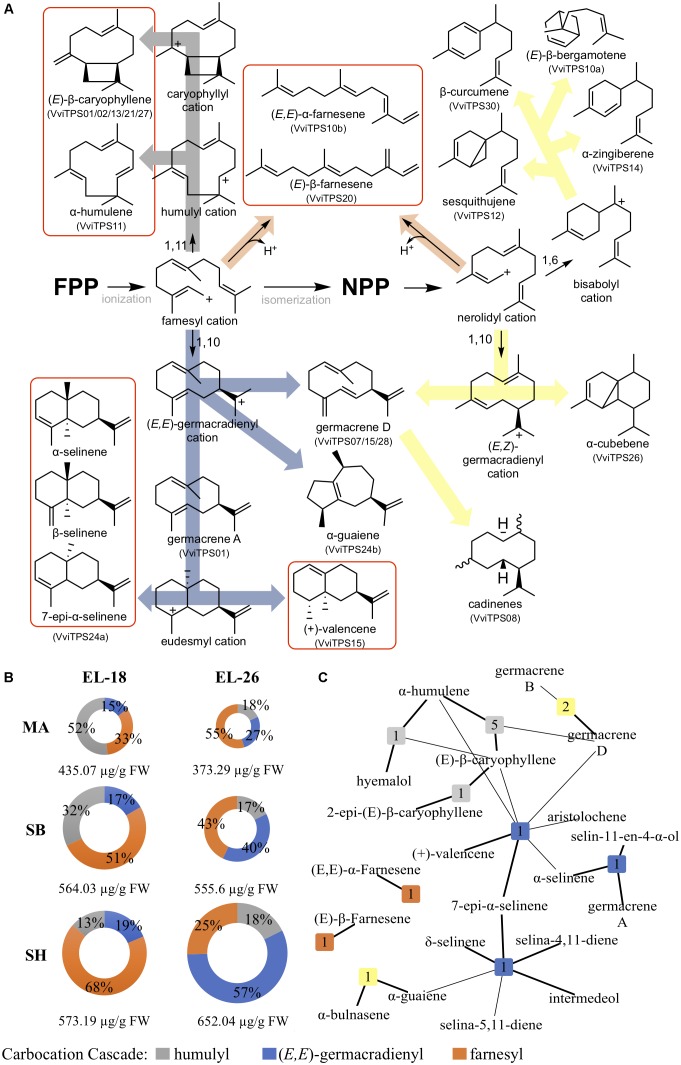
**(A)** Proposed carbocation cascades leading to cultivar-specific flower chemotypes proceed from the farnesyl cation toward the linear farnesenes (orange arrow) or through the humulyl (gray arrow) and (*E,E*)-germacradienyl (blue arrow) cations, respectively, with major end-point sesquiterpenes observed in this study shown in red squares. Yellow arrows indicate cascades for which grapevine has the biosynthetic potential (based on known functional enzymes) but not observed in the flowers studied. *VviTPS* gene models that have been linked to a functional enzyme synthesizing the respective sesquiterpenes are indicated in parentheses. **(B)** Biosynthetic flux as a percentage of the total observed sesquiterpenes for MA, SB and SH flower sesquiterpenes at two flower stages proceed through carbocation cascades where either farnesyl (orange), humulyl (gray) or (*E,E*)-germacradienyl (blue) cations serve as branchpoint intermediates. The total concentration of sesquiterpenes in μg/g FW is shown in the center of the doughnut charts. **(C)** Heterologously characterized sesquiterpene synthases that with products observed in the flowers show that there are numerous enzymes that contribute to specific sesquiterpenes. Products contributing to more than 10% of products synthesized in the heterologous expression assay are indicated by a thicker edge. Nodes are colored according to the dominant cascade that will result in the associated products.

By identifying a likely carbocation cascade required to synthesize flower sesquiterpenes, a cultivar-specific prevalence for carbocation intermediates was observed, illustrated in Figure 6. Flux through the (*E*)-humulyl cation (gray cascade) toward humulenes and caryophyllenes was consistent between stages for each cultivar. MA directs terpene biosynthesis through the farnesyl cation in both stages due to the prevalence of linear farnesene type sesquiterpenes emitted. MA, however, produced much lower total levels of sesquiterpenes in both flower stages (Figure 6B).

A trend of increased farnesene biosynthesis as flower development progressed was seen in all three cultivars (Figure 6B) with farnesene levels increasing by more than 10% from EL-18 to EL-26 in SH and SB. In these cultivars, a proportional decrease in cyclized sesquiterpenes, proceeding through the (*E,E*)-germacradienyl cation was observed. Characterized enzymes and their associated products in the context of the carbocation cascades are shown in Figure 6A. Based on the carbocation cascades that only utilize FPP, an enzyme-function network was created (Figure 6C) to illustrate the biosynthetic potential of characterized grapevine sesquiterpene synthases and how they could contribute to flower chemotypes. Nodes were numbered according to the number of gene models that transcribed an enzyme with identical function. The functional relatedness of these enzymes was represented by edges that connected products synthesized by different enzymes, with major products, <10% of contribution, shown with a thicker edge.

## Discussion

### Grapevine Flowers Are Hotspots for *VviTPS* Expression and Terpene Production

*VviTPS* gene were found to be underrepresented in previous annotations. The remapping of probes to curated gene models allowed for analysis of the VviTPS family as presented in the VCost.v3 annotation (Canaguier et al., 2017). Although we present data here only for the grapevine gene atlas, the mapping provided can be applied for analyses on the Nimblegen 090918 Vitus HX12 platform. *In silico* expression patterns (Figure 1) showed that flower development and flowering were hotspots for *VviTPS* genes, with probes associated with *VviTPS-a* and -*b* transcripts (sesqui- and monoterpene synthases), showing high relative expression during the progression from inflorescence structure differentiation (EL-14) to flower bloom (EL-23) and specific transcripts localizing to these stages. It was expected to see high *VviTPS* expression in flower tissues as it was previously identified as potential organs for VviTPS biomarkers (Fasoli et al., 2012). These biomarkers were, however, based on computational gene models of the CRIBI.v1 annotation. The *in silico* expression profiles presented in Figure 1 and Supplementary Figure 2 therefore shows the expression patterns of corrected VviTPS gene models reported by Martin et al. (2010). Although, we could refine the number genes expressed through an *in silico* re-mapping of probes to the VviTPS gene family we still found a high number of genes could not be accurately analyzed due to the observed number of ambiguous probes. Nevertheless, we clearly showed that mono- and sesquiterpene synthases are upregulated during flowering.

Volatile profiling of flowers, however, only showed high levels of sesquiterpenes with a unique major volatile for the respective cultivars (Figure 2). Previous studies showed that (+)-valencene was the major terpene for flowers from red and white cultivars with only two cultivars showing slightly higher levels of β-caryophyllene (Buchbauer et al., 1994a,b, 1995; Martin et al., 2009). The only exception was that of Muscat Bianco where monoterpenes contributed to 20% of the total flower volatiles, with sesquiterpenes contributing less then 1% (Matarese et al., 2014). (*E,E*)-α-farnesene was the second highest sesquiterpene at 22.2% for Cabernet Sauvignon flowers (Martin et al., 2009) with other cultivars showing a total contribution of 2.2% or less (Buchbauer et al., 1994a,b, 1995). The three cultivars profiled in this study emitted a unique major sesquiterpene, with the blend of volatiles emitted consisting of the same compounds, but at different ratios. Furthermore, the initial volatile screen of nine cultivars (Supplementary Figure 3) suggests even greater chemotypic differences exist.

The lack of glandular structures in domesticated grapevine (Ma et al., 2016), an accumulation of sesquiterpene transcripts and concordant emissions in flowers (Martin et al., 2009), suggests that expression and emission are linked. Martin et al. (2009) showed sesquiterpene emissions were localized to the anthers. Localization of the VvValCS protein to lipid bodies in microspores of the pollen grain, preceded by an accumulation of *VvValCS* transcripts, suggested that sesquiterpene biosynthesis was confined to male parts of the hermaphroditic flower (Martin et al., 2009), but it is not yet clear if all cultivars synthesize sesquiterpenes in this manner. Our *in silico* analysis of the VviTPS family is to some extent in agreement with the aforementioned observation. However, the lack of substantial monoterpenes volatiles in grapevine flowers, except for Muscat Bianco, suggests that there are aspects of flower terpene metabolism that likely retain monoterpenes in a non-volatile form. This has indeed been shown to be true in grape berries where yeast and/or plant glycosidases release monoterpenes during vinification (Loscos et al., 2007; Martin et al., 2012; Yauk et al., 2014).

### Isolation, Characterization, and Functional Analysis of *VvivTPS* Genes Provided Insight Into Genotypic Differences Potentially Impacting Sesquiterpene Production

Vegetative propagation and domestication of grapevine (Myles, 2013) resulted in a SNP ratio that is 2–3 higher than Arabidopsis (Martinez-Zapater et al., 2010). Profiling of these SNP differences in 5,000 germplasm accessions revealed two general domestication paths where aromatic varieties, commonly associated with table grapes, originate from Muscat or Riesling parents and less aromatic varieties used for making wine originating from the Traminer variety (Myles et al., 2011). Recently it was shown that vegetative propagation also allows for the maintenance of aberrant genome scale events where large regions of a genome can be lost due to chromosome breaking which also results widespread recombination events (Carbonell-Bejerano et al., 2017). These genome scale events have been linked to structural events that alter berry color due to deletions of hemizygous genes (Carbonell-Bejerano et al., 2017). Furthermore, evidence of genome wide transposable elements (Carrier et al., 2012), especially around the VviTPS members (Martin et al., 2010), indicate that domestication and propagation of grapevine resulted in cultivar and/or clone specific genetic changes. Linking these genotype specific structural variations with an observable phenotype presents a challenge but can be addressed to some extent when computational chemistry, functional biology, bioinformatics and chemical profiling is utilized in combination to understand enzyme mechanisms.

Phylogenetic similarity is thought to be an inaccurate predictor for function due to the effect that subtle amino acid changes have on TPS function (Yoshikuni et al., 2006), a fact that is exacerbated by the heterozygosity of grapevine and high level of duplications within the *VviTPS* family (Martin et al., 2010). Previous studies have used sequence phylogeny to establish the evolution of TPS in plants (Bohlmann et al., 1998). Various studies on the active site of sesquiterpene synthases, however, suggests that phylogenetic similarity in this region will allow for a more focused analysis by identifying amino acid residues that correlate with conserved enzyme mechanisms (Degenhardt et al., 2009; Wymore et al., 2011). These insights were recently applied in a sequence-based analysis of 262 experimentally characterized plant sesquiterpene synthases; resulting in the identification of conserved amino acid residues and motifs (Durairaj et al., 2019). Incorporating experimental evidence with the amino acid composition in the active side subsequently allowed for grouping enzymes based on carbocation intermediates utilized to produce the observed end-point sesquiterpenes (Durairaj et al., 2019). By studying the genotypic differences of selected sesquiterpene synthases from three cultivars we identified subtle sequence variations that could impact enzyme function. Although all cultivars produced a transcript for the targeted genes, structural variations resulted in many of these transcripts being non-functional (Supplementary Table 3). SNPs resulted in premature stop codons for five of the isolates (Supplementary Table 3) in this study with intron retention and partial duplication also shown (Figure 4). These structural variations effectively eliminate the targeted genes from contributing to the flower chemotype. Extrapolating these results to the extensive genotypic variation within *V. vinifera* furthermore highlights the limitations of a one-size-fits-all reference genome.

The database of plant sesquiterpene synthases (Durairaj et al., 2019) allowed us to predict the reaction mechanisms for VviTPS involved in flower sesquiterpene biosynthesis. We characterized the sequence space of the five targeted gene models by utilizing the aforementioned database in order understand how the observed genotypic variations influences enzyme function. Furthermore, we extrapolate these findings, in combination with known functional VviTPSs, to the observed flower chemotypes, illustrated in Figure 6. VviTPS10 served as a prime example for genotypic differences influencing flower chemotypes. The gene space of the three cultivar variants shows that the SB and SH variants of VviTPS10 contain premature stop codons with the latter also retaining some introns. This gene model was previously characterized as VvGWaBer synthase (VviTPS10a in Figure 6A), isolated from Gewürztraminer, producing bergamotene as major product (Martin et al., 2010). The SB and SH non-functional VviTPS10 variants showed high homology with this gene. The MA variant was, however, unique in both sequence and function with *in vivo* and *in planta* characterization resulting in (*E*)-β-farnesene as a single product. No sesquiterpene volatiles that will require isomerisation of FPP to NPP were observed. Nevertheless, a genetic capacity to synthesize NPP derived products is present in grapevine, shown by the yellow cascade of Figure 6A.

Observed flower volatiles can be grouped based on the carbocation intermediates required for their production. This allowed us to identify the cultivar specific flux from FPP with known grapevine sesquiterpene synthases producing these volatiles indicated in the cascades (Figure 6A,B). Cyclization of FPP was observed to be that first branch point with the majority of known grapevine sesquiterpene synthases proceeding though either 1,11 or 1,10 ring closures. Based on the observed flower sesquiterpenes we showed that 14.7–18.6% of FPP is directed through a 1,11-closure (gray cascade) toward humulenes and caryophyllenes with seven gene models linked with enzymes that perform this as a primary mechanism (Figure 6C). Cyclization resulting in the (*E,E*)-germacradienyl cation will be required to account for the majority of sesquiterpenes observed in SB and SH (Figure 6B), with VviTPS24 and -15 characterized to produce selinenes and (+)-valencene, respectively (Lücker et al., 2004; Martin et al., 2009, 2010). A Shiraz allelic variant of the VviTPS24 gene model resulted in the characterization of VvGuaS (indicated as VviTPS24b in Figure 6A), producing α-guaiene. This sesquiterpene serves a precursor for rotundone, which is linked to the peppery aroma profile of Shiraz wine (Siebert et al., 2008; Huang et al., 2014, 2015). Although this metabolite is not observed in flowers it serves as an example of genotypic variation impacting on terpene metabolism in a cultivar specific manner. A single amino acid difference between these allelic variants was identified as the mechanistic switch leading to either selinenes or α-guaiene (Drew et al., 2015).

The production of linear farnesenes are facilitated by enzymes that have an active site cavity where cyclization of this cation is prevented by early deprotonation of the substrate (Deligeorgopoulou and Allemann, 2003). Deprotonation of the farnesyl cation will result in stereoisomers of farnesene with VvivMATPS10 and VvCSaFar (VviTPS20) producing those in the *E* orientation. The presence of (*E,Z*)-α-farnesene in SB and SH at the flower bloom stage indicates the presence of a yet to be characterized enzyme that utilizes NPP as substrate with the nerolidyl cation being deprotonated. This novel variant of VviTPS10 presented an interesting scenario due to the extensive amino acid differences between VvGWaBer (Martin et al., 2010) and VvivMATPS10. The observed sequence differences around the active site and the distinct lineage of MA (Myles et al., 2011) suggested that VvivMATPS10 might be unique to MA, rather than a cultivar variant of VviTPS10. Southern blots targeting VviTPS10 show numerous hybridizations, suggesting the presence of multiple genomic regions homologous to VviTPS10. Probe re-mapping showed that the VviTPS10 gene model shares probes with two other gene models linked to functional enzymes (VviTPS12 and -14) (Martin et al., 2010), supporting this observation. Although each of these enzymes were unique in function it was curious to see that they shared minor products that would require a reaction mechanism proceeding through the bisabolyl carbocation (yellow cascade in Figure 6A), suggesting a degree of mechanistic conservation (Hong and Tantillo, 2014). Preliminary insight from the phased diploid Cabernet Sauvignon assembly (Chin et al., 2016; Minio et al., 2019) suggests that four homologous loci exists for VviTPS10 (Figure 5). This genome is presently being assembled and once chromosome assemblies are accessible, we should be able to elucidate if VviTPS10 represents a gene duplicated on both alleles. Nevertheless, the presence of these four homologs gives credence to our belief that VvivMATPS10 encoded by a different locus to VvGwaBer.

## Conclusion

The domestication history of grapevine has resulted in a high level of variation for *VviTPS* genes with the inbred near homozygous reference genome masking this complexity. Grapevine sesquiterpene biosynthesis was shown to differ in flowers of commercial grapevine cultivars with functional analyses of the gene space for five sesquiterpene synthases, in three cultivars, highlighting the extent of genotypic variation and the impact on floral chemodiversity. The current sesquiterpene biosynthetic landscape in *V. vinifera* suggests that there are mechanistic switches, dictated by cultivar-specific genes or variants, that allow for chemotypic differences between linear sesquiterpenes and cyclizations of FPP/NPP. The genetic potential of the respective cultivars (i.e., genotypic variation) presents multiple potential cascades toward flower sesquiterpenes with current knowledge applied to model these cascades, notwithstanding metabolic flux toward the substrate or terpene modifying enzymes. The current limitations of the reference genome for studying cultivar- and clone specific phenotypic differences is being addressed by utilizing new sequencing and assembly technologies. The phased-diploid assemblies of Cabernet Sauvignon (Chin et al., 2016; Minio et al., 2019) and 16 individual Chardonnay clones (Roach et al., 2018) will shed light on the extent of structural variations within specialized gene families across cultivars and clones as well as allelic differences within a cultivar. It is likely much more complex than what we see in the reference genome and a pangenomic view will be required in order to annotate this gene family more comprehensively.

## Author Contributions

SS, MV, and PY conceptualized the study. SS performed all field sampling, molecular biology, chemical analysis, data integration, bioinformatics, and statistical analyses. PY transformed the heterologous yeast used in functional characterization and designed the primers for VviTPS isolation. SS and PY drafted the initial manuscript. All authors contributed to the final manuscript.

## Conflict of Interest Statement

The authors declare that the research was conducted in the absence of any commercial or financial relationships that could be construed as a potential conflict of interest.
