# Supplementary material for: Linking Terpene Synthases to Sesquiterpene Metabolism in Grapevine Flowers
Source: Front Plant Sci. 2019 Feb 21;10:177. doi: 10.3389/fpls.2019.00177 (PMC6393351; doi:10.3389/fpls.2019.00177)
Supplement: Supplementary file 1 [file Table_1.DOCX]

| Gene Model | Primer (5’-3’) with restriction site modification in lowercase | | Restriction Site |
| --- | --- | --- | --- |
| VviTPS01 | Fwd | ggatccATGTCTATTCAAGTCTCAACGTGT | *Bam*HI |
|  | Rev | gtcgacTCATACTATGGGGTCAATGAGC | *Sal*I |
| VviTPS02 | Fwd | tctagaATGTCTACTCAAGTCTCAGAATGTCCT | *Xba*I |
|  | Rev | gtcgacTCATATTATGGGGTCAATGAGC | *Sal*I |
| VviTPS10 | Fwd | ggatccATGGCCTTAATTCTCGCTAC | *Bam*HI |
|  | Rev | gtcgacTCATATTGGCACAGGGTCTAT | *Sal*I |
| VviTPS27 | Fwd | ggatccATGTCTGTTCAGTCTTCAGTGG | *Bam*HI |
|  | Rev | gtcgacTCATATTGGCACAGAATCTATAAG | *Sal*I |
| VviTPS28 | Fwd | ggatccATGTCTGTTCAGTCTTCAGTGG | *Bam*HI |
|  | Rev | gtcgacTCATATTGGCACAGGATCAA | *Sal*I |

**Supplementary Table 1.** Primer sequences used to isolate targeted gene model
