# Supplementary material for: Linking Terpene Synthases to Sesquiterpene Metabolism in Grapevine Flowers
Source: Front Plant Sci. 2019 Feb 21;10:177. doi: 10.3389/fpls.2019.00177 (PMC6393351; doi:10.3389/fpls.2019.00177)
Supplement: Supplementary file 2 [file Table_2.DOCX]

**Supplementary Table 2.** Sesquiterpene composition of *V. vinifera* flowers for Muscat of Alexandria (MA), Sauvignon Blanc (SB) and Shiraz (SH) cultivars at EL-18 and EL-26. Compounds are shown as µg/g fresh weight (± SD) of four biological repeats. Compounds marked with an asterisks (*) were identified and quantified using authentic standards. Likely parent carbocation associated with each sesquiterpene is indicated by the triangles and coloured according to the cascades of Figure 6.

|  |  |  | EL-18 | | | | | | | | | | |  | EL-26 | | | | | | | | | | | |
| --- | --- | --- | --- | --- | --- | --- | --- | --- | --- | --- | --- | --- | --- | --- | --- | --- | --- | --- | --- | --- | --- | --- | --- | --- | --- | --- |
| SESQUITERPENE |  |  | MA | | |  | SB | | |  | SH | | |  | MA | | |  | SB | | |  | SH | | |  |
| β-caryophyllene* | ▲ |  | 28.61 |  | (± 1.15) |  | 32.64 |  | (± 1.14) |  | 38.84 |  | (± 2.32) |  | 27.95 |  | (± 0.5) |  | 34.27 |  | (± 2.29) |  | 41.73 |  | (± 3.2) |  |
| (+)-Aromadendrene | ▲ |  | - | | |  | 26.74 |  | (± 0.41) |  | 28.34 |  | (± 0.46) |  | - | | |  | - | | |  | 27.98 |  | (± 0.69) |  |
| α-humulene* | ▲ |  | 39.52 |  | (± 0.32) |  | 39.96 |  | (± 0.21) |  | 41.03 |  | (± 0.33) |  | 39.3 |  | (± 0.13) |  | 40.11 |  | (± 0.32) |  | 41.26 |  | (± 0.52) |  |
| (*E*)-β-farnesene* | ▲ |  | 178.19 |  | (± 45.33) |  | 55.03 |  | (± 2.5) |  | 37.11 |  | (± 0.68) |  | 132.03 |  | (± 18.94) |  | 57.53 |  | (± 7.81) |  | 44.49 |  | (± 2.1) |  |
| β-selinene | ▲ |  | - | | |  | 33.65 |  | (± 1.41) |  | 39.15 |  | (± 2.02) |  | - | | |  | 31.43 |  | (± 1.67) |  | 39.01 |  | (± 2.24) |  |
| (*E*)-caryophyllene | ▲ |  | - | | |  | 28.69 |  | (± 0.61) |  | 31.84 |  | (± 1.13) |  | - | | |  | 27.77 |  | (± 0.77) |  | 31.89 |  | (± 0.81) |  |
| (+)-valencene* | ▲ |  | 47.69 |  | (± 6.3) |  | 92.4 |  | (± 10.44) |  | 144.03 |  | (± 18.6) |  | 37.3 |  | (± 2.13) |  | 73.95 |  | (± 13.5) |  | 141.28 |  | (± 18.67) |  |
| α-selinene | ▲ |  | 32.99 |  | (± 1.84) |  | 32.01 |  | (± 1.3) |  | 38.96 |  | (± 1.95) |  | 28.34 |  | (± 0.82) |  | 30.04 |  | (± 1.24) |  | 37.08 |  | (± 1.08) |  |
| aromadendrene | ▲ |  | 29.63 |  | (± 2.2) |  | 42.11 |  | (± 2.04) |  | 27.8 |  | (± 1.15) |  | - | | |  | 26.74 |  | (± 0.14) |  | - | | |  |
| (*Z,E*)-α-farnesene | ▲ |  | - | | |  | - | | |  | - | | |  | - | | |  | 47.25 |  | (± 4.49) |  | 36.01 |  | (± 1.66) |  |
| 7-epi-α-selinene | ▲ |  | 45.53 |  | (± 5.7) |  | 83.82 |  | (± 9.27) |  | 129.54 |  | (± 15.94) |  | 36.34 |  | (± 2.07) |  | 68.21 |  | (± 11.54) |  | 126.34 |  | (± 15.92) |  |
| (*E,E*)-α-farnesene | ▲ |  | 62.54 |  | (± 14.58) |  | 139.09 |  | (± 13.39) |  | 44.35 |  | (± 3.61) |  | 72.03 |  | (± 9.36) |  | 145.04 |  | (± 31.86) |  | 84.97 |  | (± 10.2) |  |
