# Supplementary material for: Linking Terpene Synthases to Sesquiterpene Metabolism in Grapevine Flowers
Source: Front Plant Sci. 2019 Feb 21;10:177. doi: 10.3389/fpls.2019.00177 (PMC6393351; doi:10.3389/fpls.2019.00177)
Supplement: Supplementary file 4 [file Data_Sheet_1.zip › Data Sheet 1/Supplementary Data Sheet 1. Cytoscape network file with VviTPS probe remapping.pdf]

Supplementary Data Sheet 1. Cytoscape network file with VviTPS probe remapping
