# Supplementary material for: Linking Terpene Synthases to Sesquiterpene Metabolism in Grapevine Flowers
Source: Front Plant Sci. 2019 Feb 21;10:177. doi: 10.3389/fpls.2019.00177 (PMC6393351; doi:10.3389/fpls.2019.00177)
Supplement: Supplementary file 5 [file Data_Sheet_2.pdf]

Supplementary Data Sheet 2. Multiple sequence alignments (MSA) of putatively functional cultivar variants against protein sequences derived from the gene model and known functional protein(s).

```
VvGw38F3 (GerA)        -------------------------MSIQVSTCPLVQIPKPEHRPMAEFHPSIWGDQFIA 35
VvivMATPS01            -------------------------....................V.............. 35
VvivSBTPS01            -------------------------................................... 35
VvivSHTPS01            -------------------------....................V...X.......... 35
VviTPS01 (predicted)   MELAKLFRSYLPIHHLGCSIIVPSS................................... 60

VvGw38F3 (GerA)        YTPEDEDTRACKEKQVEDLKAEVRRELMAAAGNPAQLLNFIDAVQRLGVAYHFEREIEES 95
VvivMATPS01            ....................E..........D..S......................... 95
VvivSBTPS01            ..................................P......................... 95
VvivSHTPS01            ....................E..........D..S......................... 95
VviTPS01 (predicted)   ............................................................ 120

VvGw38F3 (GerA)        LQHIYDRFHDADDTEDDLYNIALQFRLLRQQGYNISCGIFNKFKDEKGSFKEDLISNVQG 155
VvivMATPS01            .........Y....D.....V....................................... 155
VvivSBTPS01            .........................................................I.. 155
VvivSHTPS01            .........................................................I.. 155
VviTPS01 (predicted)   ............................................................ 180

VvGw38F3 (GerA)        MLGLYEAAHLRVHGEDTLEEALAFTTTHLKATVESLGYPLAEQVAHALKHPIRKGLERLE 215
VvivMATPS01            ............................................................ 215
VvivSBTPS01            ................I........................................... 215
VvivSHTPS01            ................I..............A............................ 215
VviTPS01 (predicted)   ............................................................ 240

VvGw38F3 (GerA)        ARWYISLYQDEASHDKTLLKLAKLDFNLVQSLHKEELSNLARWWKELDFATKLPFARDRF 275
VvivMATPS01            ....................................Q....................... 275
VvivSBTPS01            ............................................................ 275
VvivSHTPS01            ...............................P............................ 275
VviTPS01 (predicted)   ............................................................ 300

VvGw38F3 (GerA)        VEGYFWTLGVYFEPQYSRARRILTKLFAMASIIDDIYDAYGTLEELQPFTEAIERWDIKS 335
VvivMATPS01            .........A................................................N. 335
VvivSBTPS01            ...........................S................................ 335
VvivSHTPS01            ...........................S................................ 335
VviTPS01 (predicted)   .................C.......................................... 360

VvGw38F3 (GerA)        IDHLPEYMKLFYVTLLDLYKEIDQELEKYGNQYRVYYAKEVLKSQVRAYFAEAKWSHEGY 395
VvivMATPS01            ............................D............................... 395
VvivSBTPS01            ............................D..............................F 395
VvivSHTPS01            ............................D..............................F 395
VviTPS01 (predicted)   ........................................-................... 419

VvGw38F3 (GerA)        IPTIEEYMLVALVTSGSCILATWSFIGMGEIMTKEAFDWVISDPKIITASTVIFRLMDDI 455
VvivMATPS01            ..............A...........................G................. 455
VvivSBTPS01            ..............A............................................. 455
VvivSHTPS01            ..............A............................................. 455
VviTPS01 (predicted)   ............................................................ 479

VvGw38F3 (GerA)        TTHKFEQKRGHVASGIECYMKQYGVSEEQVYSEFHKQVENAWLDINQECLKPTAVPMPLL 515
VvivMATPS01            .......................................S................V... 515
VvivSBTPS01            ............................................................ 515
VvivSHTPS01            ............................................................ 515
VviTPS01 (predicted)   ...........................................G................ 539

VvGw38F3 (GerA)        TRVVNLSRVMDVIYKEGDGYTHVGKVMKDNIGSVLIDPIV 555
VvivMATPS01            ........................................ 555
VvivSBTPS01            ........................................ 555
VvivSHTPS01            ........................................ 555
VviTPS01 (predicted)   ........................................ 579
```

MSA 1. MUSCLE Alignment of VvivTPS01 putatively functional proteins (i.e. predicted to encode for full length ORF), functional protein associated with the VviTPS01 gene model, and predicted protein derived from the gene model. Position of the active site, stretching from the DDxxD to NSE/DTE motifs, is shown in grey.

```
VvPNECar1             MSIQVSTCRLVQIPKPENRPRAEFHPSIWGDQFIAYTPEDEVSRACKEKQVEDLKEEVRR  60
VvGwECar1             ........P........H..M....................DT............A....  60
VvivSBTPS02           ..T...E.P.....E.......K...N....L..T...K............A........  60
VvivSHTPS02           .PT...E.P.............K...N....L..T....................D....  60
VviTPS02 (predicted)  ..T...E.P.............K...N....L..T.........................  60

VvPNECar1             ELMAAAGNPSQLLNFIDAVQRLGVAYHFEREIEESLQHIYDRFHDADDTNDDLYNIALRF 120
VvGwECar1             .................................................E........Q. 120
VvivSBTPS02           ...............F..............................A............. 120
VvivSHTPS02           ............................................................ 120
VviTPS02 (predicted)  ............................................................ 120

VvPNECar1             RLLRQQGYNISCGIFNKFKDEKGSFKEDLISNIQGMLGLYEAAHLRVHGEDILEEALSFT 180
VvGwECar1             .........................................................A.. 180
VvivSBTPS02           ..................................................N......A.. 180
VvivSHTPS02           .....................E...................................A.. 180
VviTPS02 (predicted)  ..............................................I...N......A.S 180

VvPNECar1             TTHLKATVESLGYPLAEQVSHALKHPIRKGLERLEARWYISLYQDEASHDKTLLKLAKLD 240
VvGwECar1             ...................A........................................ 240
VvivSBTPS02           ......M............A....R................I.....F............ 240
VvivSHTPS02           ......K............A....R................I.....F............ 240
VviTPS02 (predicted)  ......M............A....R................I.....F............ 240

VvPNECar1             FNLVQSLHKEELSNLARWWKELGFATKLPFARDRLVEGYFWIVGVYFEPQYLWAIRILTK 300
VvGwECar1             ......................D...........F......TL........SR.R..... 300
VvivSBTPS02           ....................K.D..................................... 300
VvivSHTPS02           ...................NK.D..................................... 300
VviTPS02 (predicted)  ....................K.D..................................... 300

VvPNECar1             IIVMTTVIDDIYDAYGTLEEIKHFTEAIERWDINSIDHLPKYMKLFYVALLDVYKEIEEE 360
VvGwECar1             LFS.ASI.............LQP..........K......E.......T...L....DQ. 360
VvivSBTPS02           ........................................E................... 360
VvivSHTPS02           .................F......................E................... 360
VviTPS02 (predicted)  ........................................E.....F............. 360

VvPNECar1             MEKERHQYRVHYAIDAMKNQVRAYFAEAKWFHEQHIPTMEEYMRVALLSSGYSLLATSSF 420
VvGwECar1             L..YGN....Y..KEVL.S...........S..GY...I....L...VT..SCI...W.. 420
VvivSBTPS02           ............................................................ 420
VvivSHTPS02           ...........................................S................ 420
VviTPS02 (predicted)  .....Y........E............................S...SC........... 420

VvPNECar1             IGMGEIVSKEAFDWVISDPKIIRASTVIARFMDDMTSHKFEQERGHVASGIECYMKQCGV 480
VvGwECar1             ......MT..............T.....F.L...I.T.....K..............Y.. 480
VvivSBTPS02           .........................................................Y.. 480
VvivSHTPS02           ............................................................ 480
VviTPS02 (predicted)  ......A.........R..................K..T--------------------- 459

VvPNECar1             SEEQAYKEFHNQIVNAWMDINQECLKPTAVPMPLLTRVLNLSRVMDVIYKEGDGYTHVGK 540
VvGwECar1             ....V.S...K.VE...LG...................V..................... 540
VvivSBTPS02           ............................................................ 540
VvivSHTPS02           .................L.......................................... 540
VviTPS02 (predicted)  ------R........V............................................ 513

VvPNECar1             VMKDNIGSVLIDPII 555
VvGwECar1             ..............V 555
VvivSBTPS02           ............... 555
VvivSHTPS02           ............... 555
VviTPS02 (predicted)  ..E............ 528
```

MSA 2. MUSCLE Alignment of VvivTPS02 putatively functional proteins (i.e. predicted to encode for full length ORF), functional proteins associated with the VviTPS02 gene model, and predicted protein derived from the gene model. Position of the active site, stretching from the DDxxD to NSE/DTE motifs, is shown in grey.

```
VvGwaBer              MALILATSNRSSPAPVANPETNRRTANYQPSIWGNTFIVSHTPEDEITLAHKEQQLEDLK  60
VvivMATPS10           .......................Q...........A....N........V.E........  60
VviTPS10 (predicted)  ............................................................  60

VvGwaBer              EEVRRELMASASNPSKQLKFIDAVQRLGVAYHFEKEIEEALQNTYDNYHCIDDINDDLYD  120
VvivMATPS10           .........A...L.....................D...................H....  120
VviTPS10 (predicted)  ............................................................  120

VvGwaBer              VVLRFRLLRQQGFNISCDIFNRYTDEKGRFKESLINDAYGLLGLYEAAHLRVWEEDILDE  180
VvivMATPS10           .A....................................C.....................  180
VviTPS10 (predicted)  ............................................................  180

VvGwaBer              ALAFTTTHLKSMVEHLEYPLAAQVTHALYRPLRKGLERLEARPFMSIYQDEASHSKALLK  240
VvivMATPS10           ........................S......I............................  240
VviTPS10 (predicted)  ............................................................  240

VvGwaBer              LAKLDFNQLQSLYKKELSNILGWWKDLDFSSKLPFVRDRLVEGYFWIAIACFEPQYSYAR  300
VvivMATPS10           .......L.............R.........N.......V....................  300
VviTPS10 (predicted)  ............................................................  300

VvGwaBer              RIQTKLHALMTTTDDIFDAYGTLEELEFFTEAIGRWDIDSTHQLPEYMKPCYQAVLDAYK  360
VvivMATPS10           .........LI................LY.........N.I................FF.  360
VviTPS10 (predicted)  ............................................................  360

VvGwaBer              EIEDMENTERSHSVHQAKDAMKNLVQAYLVEAKWFHGKYIPTIEEYMRVALVSIGAPVLT  420
VvivMATPS10           ...-.Q.....YC..H.Q.........F....................D....S..T.FA  419
VviTPS10 (predicted)  ............................................................  420

VvGwaBer              FISFIGMGEIATKEVFDWLQQNPKIVRASSKVIRLMDDMATHKFEQERGHIASSIECYMK  480
VvivMATPS10           I....V............V.........L.T.M...N..........S............  479
VviTPS10 (predicted)  ............................................................  480

VvGwaBer              QHGVSEQQAYEEFHKQLENAWKDINEECLKPTAVPMLLLSRLLNFARAADVMYKGQKDEF  540
VvivMATPS10           .............................R..............LC.....V......A.  539
VviTPS10 (predicted)  ............................................................  540

VvGwaBer              THLGEVMKNNISMLLIDPVPI  561
VvivMATPS10           .....................  560
VviTPS10 (predicted)  .....................  561
```

MSA 3. MUSCLE Alignment of VvivTPS10 putatively functional proteins (i.e. predicted to encode for full length ORF), functional protein associated with the VviTPS10 gene model, and predicted protein derived from the gene model. Position of the active site, stretching from the DDxxD to NSE/DTE motifs, is shown in grey.

```
VvGwECar2              MSVQSSVVLLAPSKNLSPEVGRRCANYHPSIWGDHFLSYASEFTNTDDHLKQHVQQLKEE  60
VvivSBTPS27            ..........................F.................................  60
VvivSHTPS27            ..........................F.................................  60
VvivMATPS27            ............................................................  60
VviTPS27 (predicted)   ............................................................  60

VvGwECar2              VRKMLMAADDDSVQKLLLIDAIQRLGVAYHFESEIDEALKHMFDGSVASAEEDVYTASLR  120
VvivSBTPS27            ............................................................  120
VvivSHTPS27            ............................................................  120
VvivMATPS27            ............................................................  120
VviTPS27 (predicted)   ............................................................  120

VvGwECar2              FRLLRQQGYHVSCDLFNNFKDNEGNFKESLSSDVRGMLSLYEATHLRVHGEDILDEALAF  180
VvivSBTPS27            ..........................R.................................  180
VvivSHTPS27            ............................................................  180
VvivMATPS27            ............................................................  180
VviTPS27 (predicted)   ............................................................  180

VvGwECar2              TTTHLQSAAKYSLNPLAEQVVHALKQPIRKGLPRLEARHYFSIYQADDSHHKALLKLAKL  240
VvivSBTPS27            ..............................................G.............  240
VvivSHTPS27            ............................................................  240
VvivMATPS27            ............................................................  240
VviTPS27 (predicted)   ............................................................  240

VvGwECar2              DFNLLQKLHQKELSDISAWWKDLDFAHKLPFARDRVVECYFWILGVYFEPQFFLARRILT  300
VvivSBTPS27            ............................................................  300
VvivSHTPS27            ............................................................  300
VvivMATPS27            ............................................................  300
VviTPS27 (predicted)   ............................................................  300

VvGwECar2              KVITMTSTIDDIYDVYGTLEELELFTEAVERWDISVIDQLPEYMRVCYRALLDVYSEIEE  360
VvivSBTPS27            ............................................................  360
VvivSHTPS27            ............................................................  360
VvivMATPS27            ............................................................  360
VviTPS27 (predicted)   ............................................................  360

VvGwECar2              EMAKEGRSYRFYYAKEAMKKQVRAYYEEAQWLQAQQIPTMEEYMPVASATSGYPMLATTS  420
VvivSBTPS27            ..............................................V.............  420
VvivSHTPS27            ............................................................  420
VvivMATPS27            ............................................................  420
VviTPS27 (predicted)   ............................................................  420

VvGwECar2              FIAMGDVVTKETFDWVFSEPKIVRASATVSRLMDDMVSHKFEQKRGHVASAVECYMKQHG  480
VvivSBTPS27            ............................................................  480
VvivSHTPS27            ............................................................  480
VvivMATPS27            ............................................................  480
VviTPS27 (predicted)   ............................................................  480

VvGwECar2              ASEQETRDEFKKQVRDAWKDINQECLMPTAVPMTVLMRILNLARVMDVVYKHEDGYTHSG  540
VvivSBTPS27            ............................................................  540
VvivSHTPS27            ............................................................  540
VvivMATPS27            ............................................................  540
VviTPS27 (predicted)   ............................................................  540

VvGwECar2              TFLKDLVTSLLIDSVPI  557
VvivSBTPS27            .................  557
VvivSHTPS27            .................  557
VvivMATPS27            .................  557
VviTPS27 (predicted)   .................  557
```

MSA 4. MUSCLE Alignment of VvivTPS27 putatively functional proteins (i.e. predicted to encode for full length ORF), functional protein associated with the VviTPS27 gene model, and predicted protein derived from the gene model. Position of the active site, stretching from the DDxxD to NSE/DTE motifs, is shown in grey.

```
VvGerD                MSVQSSGVLLAPSKNLSPEVGRRCANFHPSIWGDHFLSYASEFTNTDDHLKQHVQQLKEE  60
VvivMATPS28           ......V.....................................................  60
VviTPS28 (predicted)  ......V.....................................................  60

VvGerD                VRKMLMAADDDSAQKLLLIDAIQRLGVAYHFESEIDEVLKHMFDGSVVSAEEDVYTASLR 120
VvivMATPS28           ............................................................ 120
VviTPS28 (predicted)  ............................................................ 120

VvGerD                FRLLRQQGYHVSC-DLFNNFKDNEGNFKESLSSDVRGMLSLYEATHFRVHGEDILDEALA 179
VvivMATPS28           .............A.............................................. 180
VviTPS28 (predicted)  .............-.............................................. 179

VvGerD                FTTTHLQSATKHSSNPLAEQVVHALKQPIRKGLPRLEARHYFSVYQADDSHNKALLKLAK 239
VvivMATPS28           ............................................................ 240
VviTPS28 (predicted)  ............................................................ 239

VvGerD                LDFNLLQKLHQKELSDISAWWKDLDFAHKLPFARDRVVECYFWILGVYFEPQFFFARRIL 299
VvivMATPS28           ............................................................ 300
VviTPS28 (predicted)  ............................................................ 299

VvGerD                TKVIAMTSIIDDIYDVYGTLEELELFTEAVERWDISAIDQLPEYMRVCYQALLYVYSEIE 359
VvivMATPS28           ............................................................ 360
VviTPS28 (predicted)  ............................................................ 359

VvGerD                EEMAKEGRSYRLYYAKEAMKNQVRAYYEEAKWLQVQQIPTMEEYMPVALVTSAYSMLATT 419
VvivMATPS28           ............................................................ 420
VviTPS28 (predicted)  ............................................................ 419

VvGerD                SFVGMGDAVTKESFDWIFSKPKIVRASAIVCRLMDDMVFHKFEQKRGHVASAVECYMKQH 479
VvivMATPS28           ......................................S..................... 480
VviTPS28 (predicted)  ......................................S..................... 479

VvGerD                GASEQETPNEFPQPVREAWKDINEECLIPTAVPMPILMRVLNLARVIDVIYKNEDGYTHF 539
VvivMATPS28           .......H...HKQ..D..........................................S 540
VviTPS28 (predicted)  .......H...HKQ..D..........................................S 539

VvGerD                GAVLKDFVTSMLIDPVPI 557
VvivMATPS28           .T................ 558
VviTPS28 (predicted)  .T................ 557
```

MSA 5. MUSCLE Alignment of VvivTPS28 putatively functional proteins (i.e. predicted to encode for full length ORF), functional protein associated with the VviTPS28 gene model, and predicted protein derived from the gene model. Position of the active site, stretching from the DDxxD to NSE/DTE motifs, is shown in grey.
