# Supplementary figures and images for: Linking Terpene Synthases to Sesquiterpene Metabolism in Grapevine Flowers
Source: Front Plant Sci. 2019 Feb 21;10:177. doi: 10.3389/fpls.2019.00177 (PMC6393351; doi:10.3389/fpls.2019.00177)

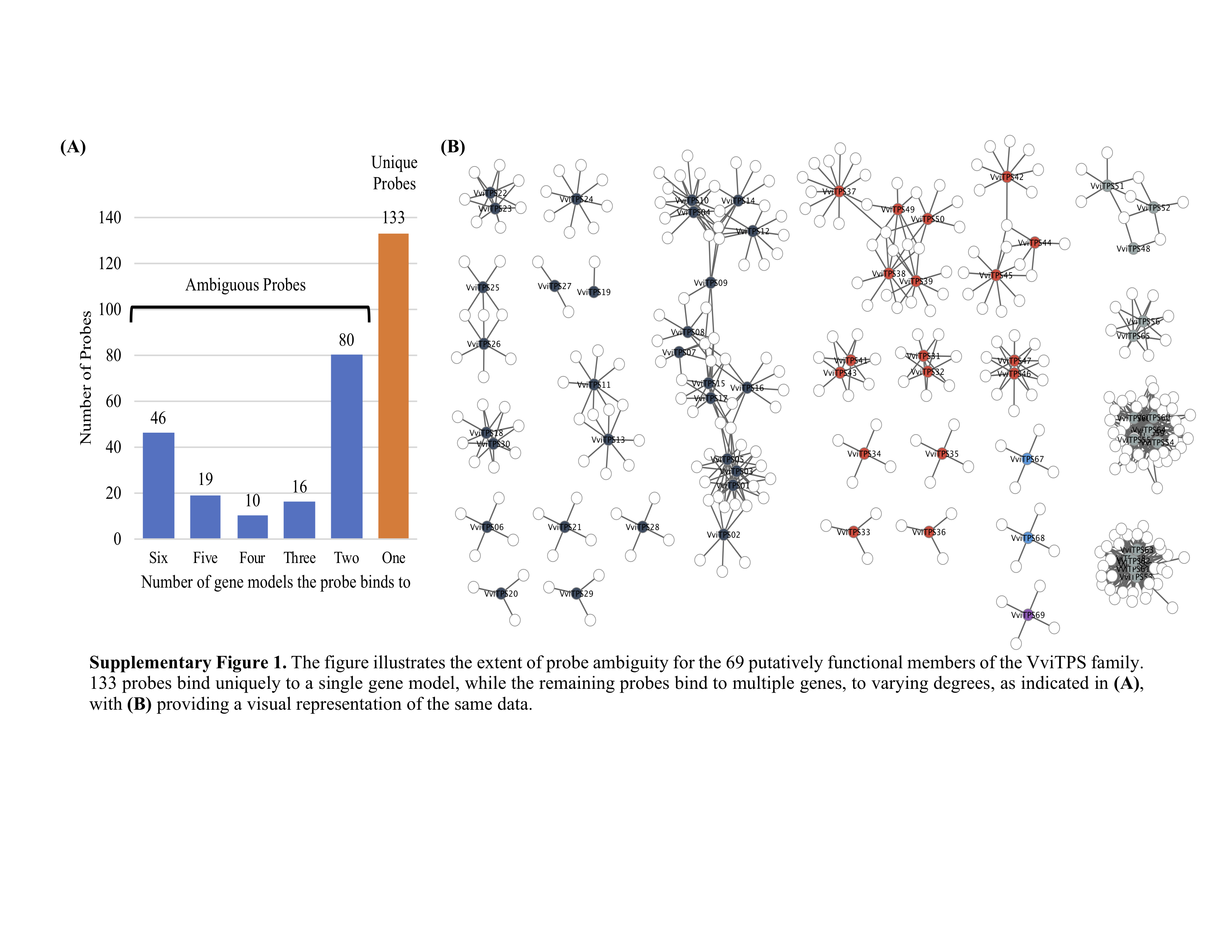

Supplement: Supplementary file 6 [file Image_1.TIFF]

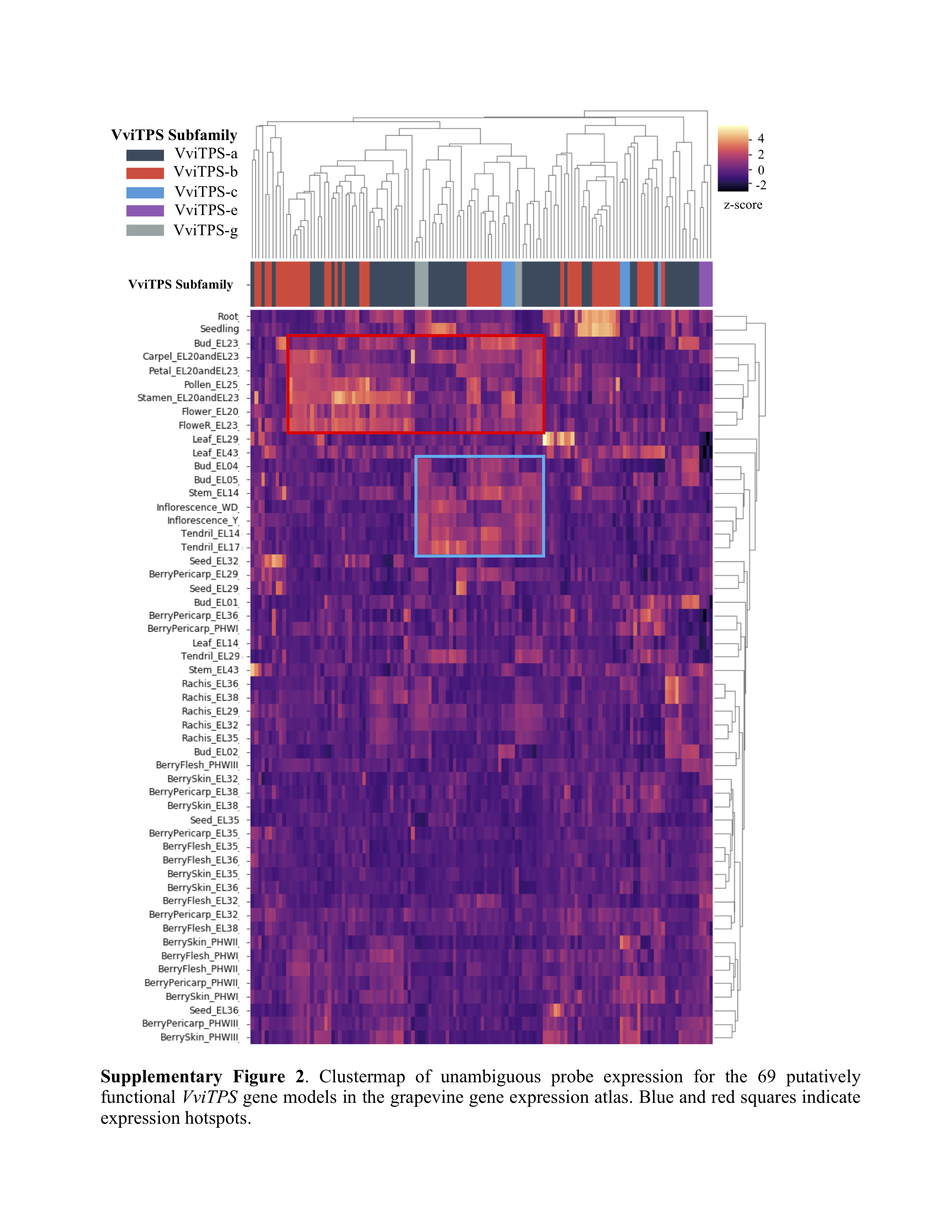

Supplement: Supplementary file 7 [file Image_2.TIFF]

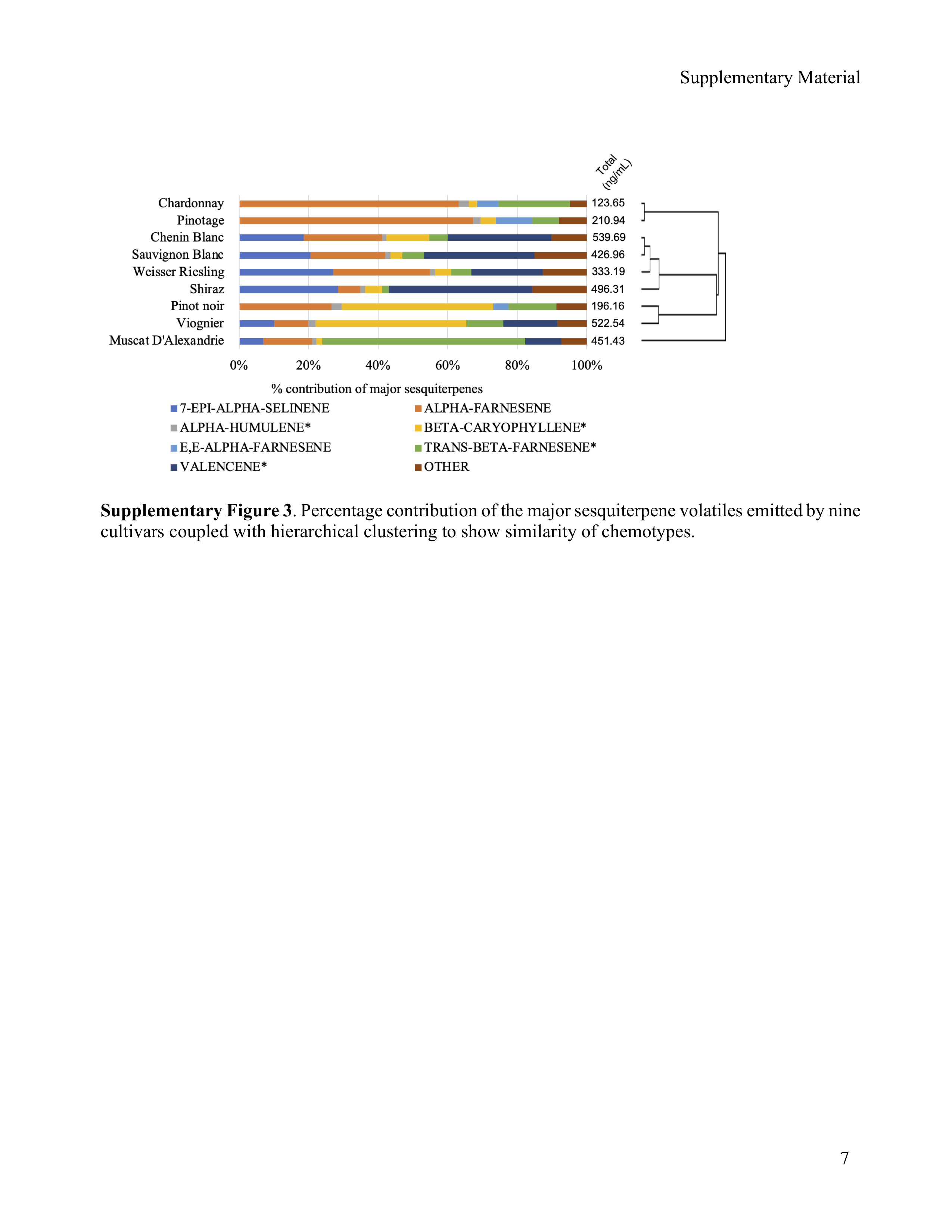

Supplement: Supplementary file 8 [file Image_3.TIFF]

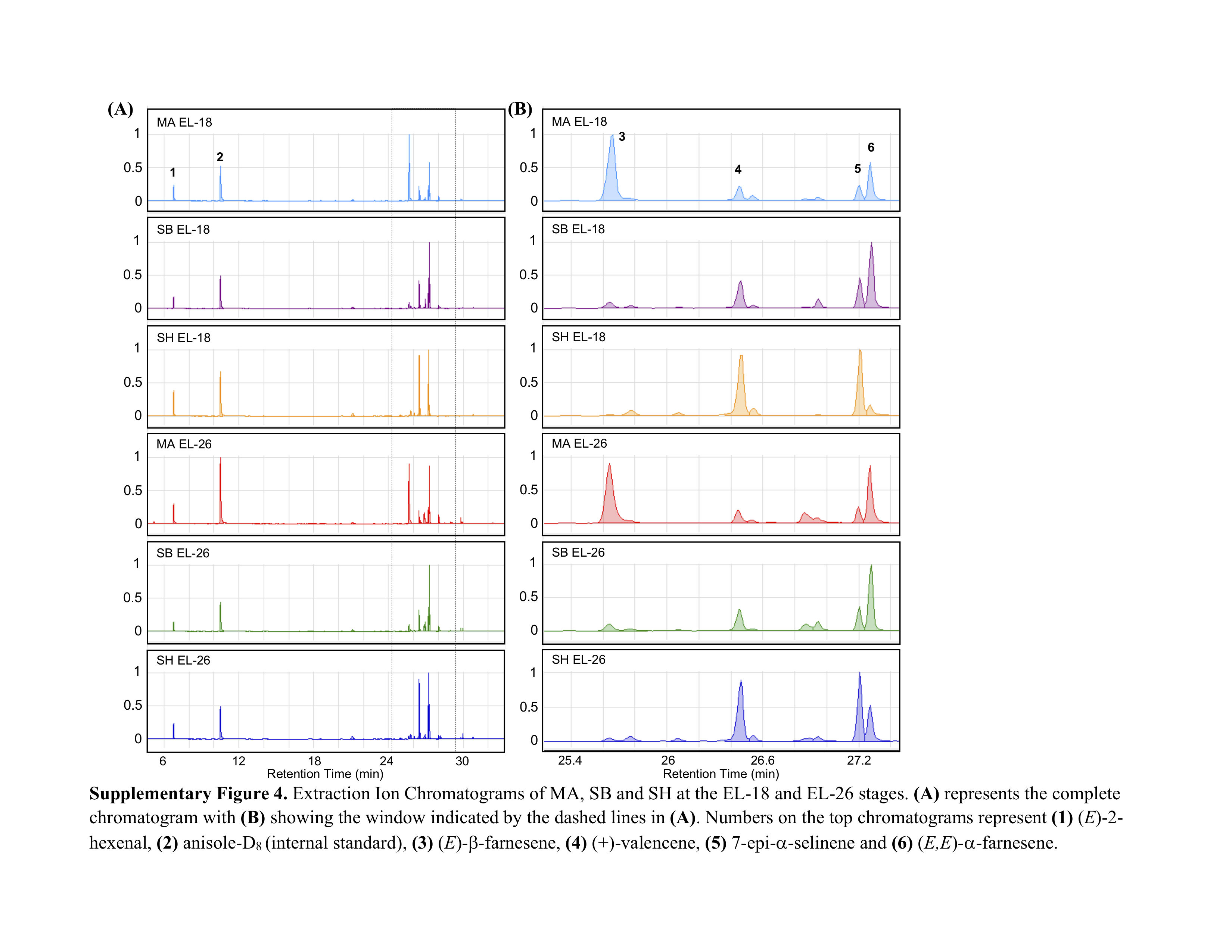

Supplement: Supplementary file 9 [file Image_4.TIFF]

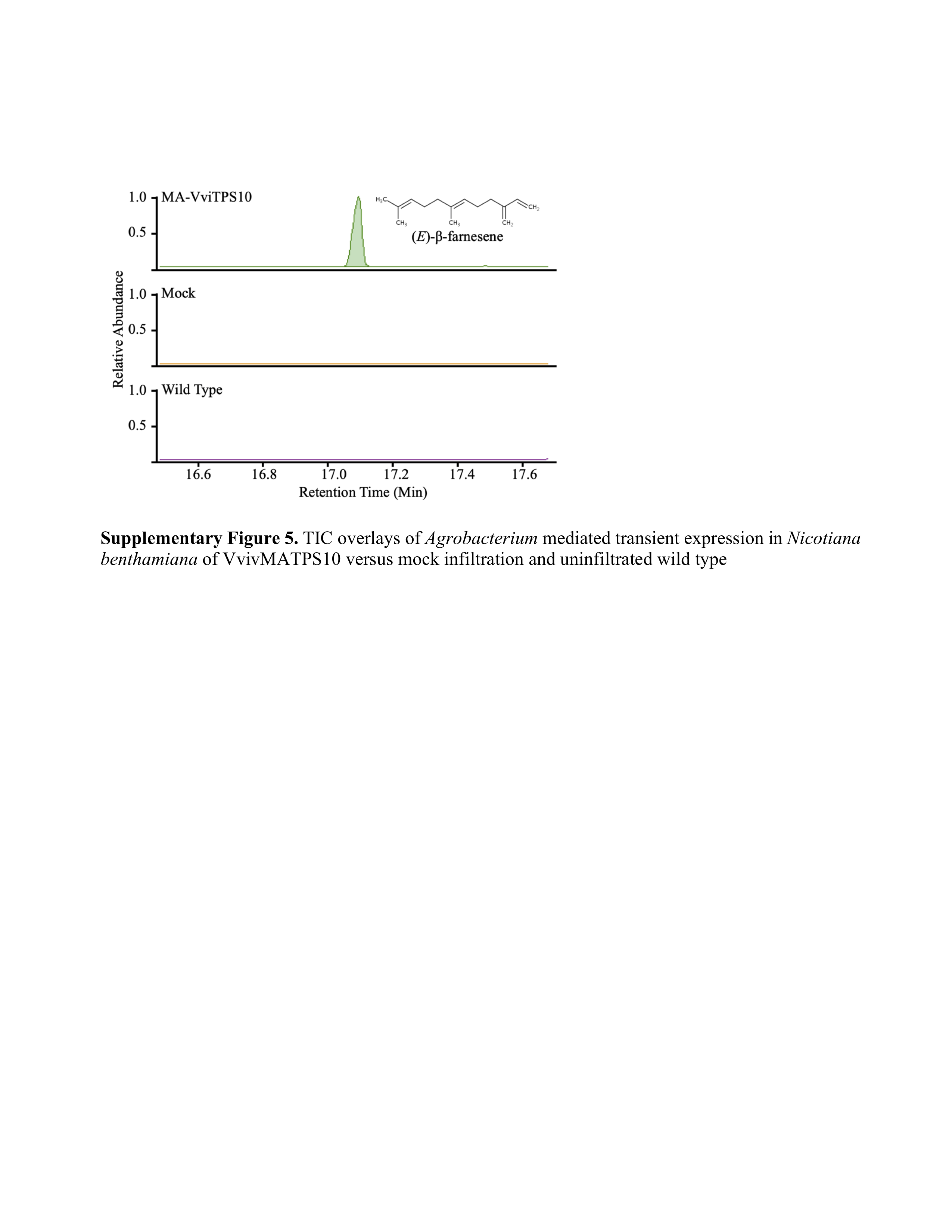

Supplement: Supplementary file 10 [file Image_5.TIFF]

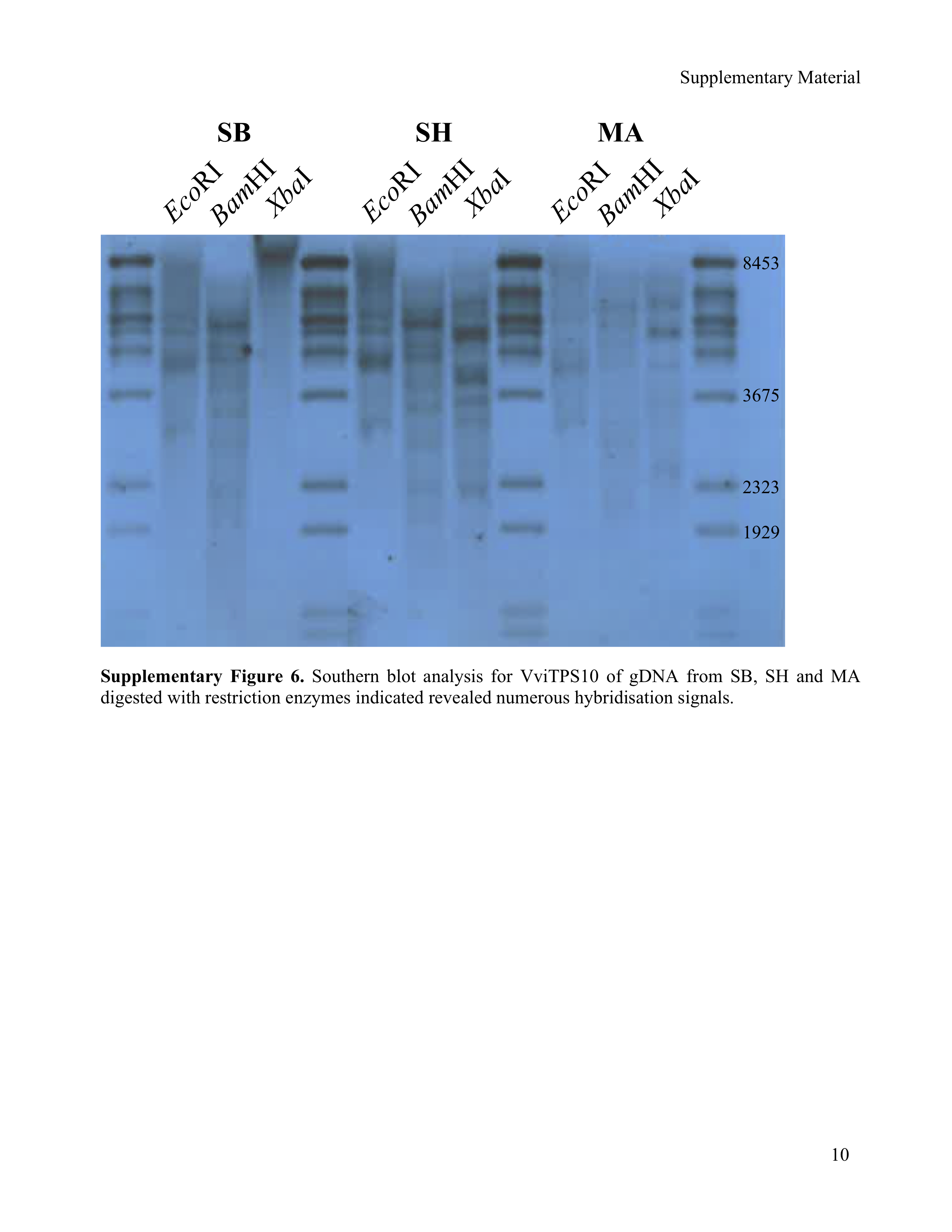

Supplement: Supplementary file 11 [file Image_6.TIFF]

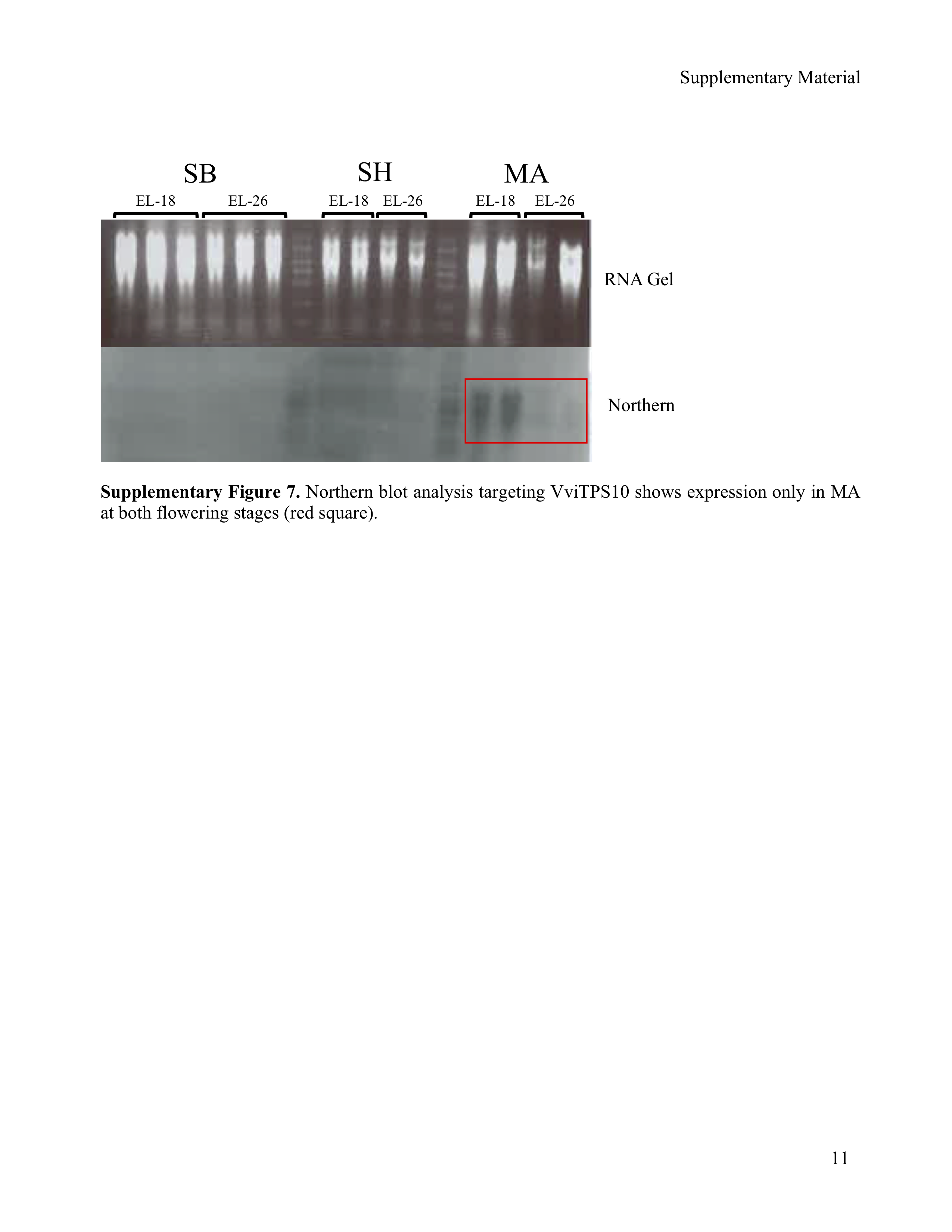

Supplement: Supplementary file 12 [file Image_7.TIFF]
